# EGFRvIII-positive glioblastoma contributes to immune escape and malignant progression via the c-Fos-MDK-LRP1 axis

**DOI:** 10.1038/s41419-025-07771-1

**Published:** 2025-06-17

**Authors:** Feng Yuan, Yingshuai Wang, Lei Yuan, Ting Tang, Lei Ye, Yan Li, Xingliang Dai, Hongwei Cheng

**Affiliations:** 1 Department of Neurosurgery, The First Affiliated Hospital of Anhui Medical University, Hefei, Anhui China; 2 Department of Neurosurgery, Affiliated Jinling Hospital, Medical School of Nanjing University, Nanjing, China; 3 Department of Internal Medicine III, University Hospital Munich, Ludwig-Maximilians-University Munich, Munich, Germany; 4 Department of Anesthesiology, The First Affiliated Hospital of Anhui Medical University, Hefei, Anhui China; 5 Department of Neurosurgery, Xuanwu Hospital Capital Medical University, Beijing, China

**Keywords:** CNS cancer, Cancer microenvironment

## Abstract

Epidermal growth factor receptor variant III (*EGFRvIII*) confers growth advantage to glioblastoma multiforme (GBM) and is associated with significantly shorter survival in GBM patients. The interaction between tumor cells and macrophages plays a crucial role in tumor development, supporting angiogenesis, nurturing tumor stem cells, and promoting immune-suppressive TME. Therefore, elucidating the potential mechanisms by which *EGFRvIII* mutation in GBM cells regulates surrounding immune cells to drive tumor progression may provide new targets for precise immune therapy for specific GBM subtypes or genotypes. In this study, we found that *EGFRvIII* was the most common form of *EGFR* mutation, with an incidence rate of 22.13% in glioma patients and 33.3% in GBM patients. Mechanistically, we found for the first time that *EGFRvIII*-positive GBM secretes high levels of MDK via the ERK-c-Fos signaling pathway. Subsequently, GBM cell-secreted MDK drives macrophage polarization towards the M2 phenotype and secretion of the cytokine CXCL1 via activation of the macrophage surface receptor LRP1 and downstream pathways. In turn, these macrophages secrete CXCL1, which attracts immune-suppressive cells and TAMs to support GBM growth. In the intracranial glioma model, blocking MDK signaling pathway could inhibit macrophage polarization towards the M2 phenotype and tumor malignant progression. In summary, our study for the first time found that *EGFRvIII*-positive GBM can drive macrophage polarization towards M2 phenotype and secretion of immune-suppressive cytokine CXCL1 via the c-Fos-MDK-LRP1 signaling pathway, providing new targets for precise immune therapy for specific GBM subtypes or genotypes.

## Introduction

Glioma is the most common primary tumor of the central nervous system (CNS), with glioblastoma multiforme (GBM) being the most malignant, accounting for 47.1% of primary CNS malignant tumors [1]. GBM is a highly lethal and poor prognosis tumor, with standard treatment consisting of surgical resection, synchronous radiotherapy and chemotherapy, adjuvant chemotherapy, and/or tumor-treating fields (TTFs). Despite the constantly evolving diagnostic and therapeutic concepts, increasingly precise surgeries, and continuous emergence of drugs for glioma, the median overall survival of GBM patients is only about 14-16 months [2]. In recent years, immunotherapy has made significant progress in the treatment of non-small cell lung cancer, melanoma, and hematological malignancies, and there is also a large amount of research on immunotherapy for glioma [3].

GBM is known for its highly inhibitory immune microenvironment, which is a key obstacle to immunotherapy [4]. On the one hand, many molecular pathways and cell functions are involved in regulating tumor immunity, and GBM cells produce effective immune inhibitory molecules, including transforming growth factor-beta (TGF-β), interleukin-10 (IL-10), and indoleamine 2,3-dioxygenase (IDO). GBM can also express immune checkpoint ligands that significantly inhibit immune responses, such as PD-L1, CTLA4, and TIM3 [5]. In addition, regulatory T cells (Tregs), M2-type tumor-associated macrophages (TAMs), and myeloid-derived suppressor cells (MDSCs) in the tumor microenvironment (TME) have been reported to be associated with poor overall survival of GBM patients [6]. On the other hand, the interaction between tumor cells and immune cells plays a crucial role in tumor development, supporting angiogenesis, nurturing tumor stem cells, and promoting immune suppressive TME [7].

Another major characteristic of GBM is its extensive heterogeneity at the cellular and molecular levels. Based on genomic abnormalities and gene expression, The Cancer Genome Atlas (TCGA) has classified GBM into four subtypes, including classical, mesenchymal, proneural, and neural subtypes [8]. Among the four subtypes, mesenchymal GBM exhibits the most abundant stromal component, most of which are TAMs, which typically display M2-type macrophages. TAMs are infiltrating immune cells derived from bone marrow and microglia cells, accounting for 30% to 50% of all cells in GBM tumors. This cell population plays a key role in GBM progression [9]. Mesenchymal GBM typically contains gene mutations in the phosphatase and tensin homolog (PTEN), TP53, and NF1, and exhibits a highly immune suppressive microenvironment, strong invasiveness, and treatment resistance compared to other subtypes [10].

Epidermal growth factor receptor (*EGFR*) is frequently overexpressed in different cancers, and its expression level is positively correlated with cancer progression and poor prognosis [11]. Different mutations of *EGFR* can lead to cancer heterogeneity, with the most common mutation being the epidermal growth factor receptor variant III (*EGFRvIII*). *EGFR* mutations are common in GBM, with the *EGFRvIII* encoding mutation rate in GBM being about 50%, and *EGFRvIII* being specifically present on 28% to 30% of GBM cells [12]. A study published in the Journal of Clinical Oncology, based on clinical data from 649 newly diagnosed GBM patients (472 with *EGFRvIII*-negative and 177 with *EGFRvIII*-positive), indicates that *EGFRvIII* serves as an independent prognostic factor, with *EGFRvIII*-positive GBM patients exhibiting a poorer prognosis. *EGFRvIII* mutations lead to the deletion of exons 2-7 in the receptor extracellular domain, resulting in constitutive activation of downstream signaling pathways, including the MAPK, PI3K/AKT, and STAT pathways [13].

In this study, we investigated the impact of *EGFRvIII* mutations in GBM cells on TAMs and how this affects macrophage polarization, as well as how infiltrating macrophages in turn support GBM growth and malignant progression. Our goal is to identify practical therapeutic targets that play a role in the tumor cell-macrophage interaction in the GBM TME with *EGFRvIII* mutations.

## Materials and methods

### Databases and samples

One publicly available (GSE141383) human GBM single-cell RNA sequencing (scRNA-seq) datasets were obtained from the Gene Expression Omnibus (GEO) database (http://www.ncbi.nlm.nih.gov/geo/). The GSE141383 database contains 9 GBM samples, including 2 *EGFRvIII*-positive and 4 *EGFRvIII*-negative cases.

The three databases used in this study were TCGA, Chinese Glioma Genome Atlas (CGGA), and Rembrandt. AffyU133A (n = 539) and IlluminaHiSeq (n = 702) microarray RNA-seq data from TCGA were downloaded from the University of California Santa Cruz Cancer Genomics Browser (https://genome-cancer.ucsc.edu), and relevant clinical and molecular information was obtained from the TCGA database (https://tcga-data.nci.nih.gov/docs/publications/lgggbm_2015/). CGGA mRNAseq data and clinical information were obtained from the CGGA database (http://www.cgcg.org.cn/, n = 325). Rembrandt database was obtained from the National Cancer Institute Molecular Brain Tumor Data Storage Repository (https://gdoc.georgetown.edu/gdoc) queue (n = 313).

Tissue microarrays data were collected from 85 patients with glioma who underwent craniotomy for tumor resection in the Neurosurgery Department of The First Affiliated Hospital of Anhui Medical University from December 2009 to January 2020, according to the 2016 revised WHO classification of CNS tumors, and 5 cases of non-tumor brain tissue were included as controls. In addition, 71 patients with glioma who underwent craniotomy for tumor resection in the Neurosurgery Department of The First Affiliated Hospital of Anhui Medical University from December 2017 to August 2022 were collected, all of whom received tumor molecular diagnostic testing, and technical support was provided by Genetron Health (Beijing, China); according to the 2021 revised WHO classification of CNS tumors. This study was conducted in accordance with the principles of the Helsinki Declaration. All patients provided written consent for their samples to be used for biomedical research and were approved by the Ethics Committee of The First Affiliated Hospital of Anhui Medical University (PJ2024-06-19).

### Cell lines and cell culture

Human glioma cell lines U87MG, U251MG, T98MG, and mouse glioma cell line GL261 were purchased from the Cell Resource Center of the Shanghai Institute of Life Sciences, Chinese Academy of Sciences. Glioma cells were cultured in high-glucose DMEM medium containing 10% heat-inactivated fetal bovine serum (FBS), 100 U/ml penicillin, and 0.1 mg/ml streptomycin and incubated in a constant temperature incubator at 37 °C and 5% CO2 saturation. Human THP-1 (human monocytic leukemia) cell line was kindly provided by Professor Zhang Junxia’s team from the First Affiliated Hospital of Nanjing Medical University. Human THP-1 cells were cultured in RPMI-1640 medium containing 10% heat-inactivated FBS, 100U/ml penicillin, and 0.1 mg/ml streptomycin.

### Co-culture of cells

Human glioma cells and THP-1 cells were co-cultured using Transwell chamber. The Transwell chamber was placed in a six-well plate, with a layer of PC or PET membrane between the upper and lower chambers. The membrane had micropores with a maximum diameter of 12.0um and a minimum diameter of 0.1um. When the pore size was less than 3.0um, cells could not pass through the membrane, but cytokines and other molecules could freely pass through. The steps for co-culture were as follows: (1) THP-1 cells were induced to differentiate into macrophages with 185 ng/ml Phorbol-12-myristate-13-acetate (PMA); (2) Cells were digested, centrifuged, and finally resuspended; (3) Human glioma cells and PMA-induced THP-1 cells were separately seeded in the upper and lower chambers of the Transwell, with a 1:1 ratio of cells in the upper chamber with high-glucose DMEM as the culture medium and lower chambers chamber with RPMI-1640 as the culture medium. (4) After co-culture, the cell status in the upper and lower chambers was observed under a microscope. If necessary, relevant staining experiments could be performed. Cells in the upper and lower chambers could also be collected for RNA or protein extraction to detect target genes, as well as other phenotype experiments (generally after 48–72 h of treatment).

### Single-cell RNA-seq analysis

We obtained single-cell RNA sequencing (scRNA-seq) profiles from 16,028 single cells derived from six tissue samples, including four EGFRvIII(−) glioblastoma cases and two EGFRvIII(+) glioblastoma cases. After applying quality control criteria, 14,013 single cells were retained for further analysis. To visualize the distribution of the scRNA-seq profiles, we applied t-distributed Stochastic Neighbor Embedding (t-SNE) to reduce the dimensionality of these datasets. Batch effects within these datasets were appropriately corrected using the “fastMNN” algorithm. Through unsupervised clustering, the cells were successfully grouped into 17 clusters. Based on the expression patterns of markers from the CellMarker database, we manually annotated these clusters into the following seven cell types: (1) GBM cancer cells (expressing SOX2, PARP1, and CCND2); (2) M1-type macrophages (expressing CD68, CD74, TSPO, and CD86); (3) M2-type macrophages (expressing CD68, CD74, and CD163); (4) T cells or NK cells (expressing CXCR4 and S100A4); (5) endothelial cells (expressing A2M and APOLD1); (6) astrocytes (expressing GFAP and SOX9); and (7) oligodendrocytes (expressing CNP, MBP, and PLP1). Single-cell data analysis was performed as previously described by Yuan et al. [14]. Single-cell RNA-seq analysis was performed using the “Seurat” R package (version 4.0.2).

### Lentiviral infection

Lentiviruses carrying EGFRvIII (NM_001346941.2) and MDK (NM_010784.5) were transfected. The lentiviral plasmids carrying EGFRvIII and MDK were synthesized by Shanghai GenePharma Co., Ltd and Shanghai hanyinbt Co., Ltd, respectively.

The lentivirus transfection steps were as follows: lentivirus transfection was performed when the cell fusion degree reached 80–90%. Five hundred microliters serum-free medium was added to a 12-well plate, and an appropriate volume of *EGFRvIII*/MDK lentivirus or control lentivirus was added to each well. The plate was placed in a cell culture incubator and incubated for 12 h. The medium was then replaced with complete medium, and the cells were further cultured for 48-72 h before being passaged. Stable transfected cell lines were selected using puromycin and observed under a fluorescence microscope.

### Cell transfection with siRNA

SiRNA targeting c-Fos was purchased from Shanghai GenePharma Co., Ltd. The siRNA powder was dissolved in ultrapure water ddH_2_O to prepare a 20 μM solution. The siRNA sequence for c-Fos, CXCL1 are as follows: UGGUUUACAUGUCGACUAA (control siRNA), GCGAGGAGACAGCCAAUUA (siFOS), CCAAGAACAUCCAAAGUGUTT (siCXCL1). The transfection reagent used was the jetPRIME® transfection kit from Polyplus, Germany.

The siRNA transfection process is as follows: (1) Add 2 μg of DNA to 200 μL of jetPRIME® buffer. Gently vortex to mix. (2) Add 4 μL of jetPRIME®, vortex for 10 s, and let stand. (3) Incubate at room temperature for 10 min. (4) Add 200 μL of the transfection complex to the cells containing serum. (5) Gently shake the culture dish to evenly distribute the transfection complex into the cells. (6) If necessary, replace with complete culture medium 4 h after transfection and continue to culture. (7) Transfection was analyzed after 24 h or according to experience.

### Western blotting

After completing the gel casting, SDS-PAGE was performed until the sample ran to the green line at the bottom. Then, a wet transfer method (300 mA, 90 min) was used, followed by blocking with 5% BSA for 1 h. The primary antibody, which was pre-prepared, was added and incubated overnight at 4 °C. Antibody information is shown in the Supplementary Table S1. The membrane was washed three times with TBST, and then the corresponding secondary antibody was added and incubated at room temperature for 1 h. The membrane was washed three times with TBST again. Enhanced chemiluminescence (ECL) was used for detection, and the G-box imaging system was used for exposure.

### qPCR

After RNA extraction, cDNA was synthesized using a cDNA synthesis kit (TransGen Biotech, AE311), followed by Real-time PCR amplification. The qPCR primers were designed and synthesized by Sangon Biotech (Shanghai) Co.,Ltd, and the primer sequences are shown in the Supplementary Table S2. After PCR amplification, the real-time fluorescence quantitative PCR instrument automatically analyzed the results. The 2-ΔΔCT method was used to analyze the expression differences of the target gene between the control group and each test group. The formula for calculation is as follows: ΔCt = Ct target gene - Ct internal reference, then the average value of ΔCt control group was obtained, which was denoted as ΔCt control average. The ΔCt of each group was subtracted from ΔCt control average to obtain the ΔΔCt value, that is, ΔΔCt = ΔCt sample - ΔCt control average, and then the 2−ΔΔCT value of each group was calculated, which represents the relative expression level of the gene in each group.

### Enzyme-linked immunosorbent assay (ELISA)

The Human Midkine ELISA Kit (EK1253) and Human CXCL1/GRO-α ELISA Kit (EK196) were purchased from Hangzhou Lianke Biotechnology Co., Ltd. After 48 h of cell culture, the supernatant from each group was collected. Blank wells were set up, and 100 μL of diluted supernatant samples and standards were added to the enzyme-linked immunosorbent assay (ELISA) plate, followed by incubation at 37 °C for 90 min. The plate was washed three times, and 100 μL of biotin-labeled human MDK/CXCL1 antibody was added to each well, followed by incubation at 37 °C for 60 min. After another three washes, 100 μL of streptavidin-peroxidase complex was added to each well (except the blank wells), and the plate was incubated at 37 °C for 30 min. The plate was then washed four times, and 100 μL of tetramethylbenzidine (TMB) substrate solution was added to each well, followed by a 15-min reaction at 37 °C. The reaction was stopped by adding stop solution, and the absorbance was immediately measured at a wavelength of 450 nm. The standard curve data were fitted using SPSS to generate a regression equation for concentration-absorbance, and the MDK/CXCL1 concentrations of the samples were calculated accordingly.

### Cell proliferation assay with cell counting kit-8 (CCK-8)

The CCK-8 (BS350A) was purchased from Biosharp, a brand of Lianjieke Technology Co., Ltd. First, cells were seeded into a 96-well plate and cultured until adherence. Next, different concentrations of drugs or treatment factors were added, and the cells were further cultured for a specified period. Then, CCK-8 reagent was added to each well, followed by incubation for 1.5 h to allow viable cells to reduce the reagent to an orange-yellow formazan product. Finally, the absorbance (OD value) was measured at a wavelength of 450 nm using a microplate reader, and the cell proliferation rate was calculated to evaluate cell viability and drug effects.

### High-throughput sequencing

The preparation of transcriptome and proteome libraries and sequencing process were performed by LC-Bio Technology CO., Ltd (Hangzhou, China). Total RNA was extracted using TRIzol (Invitrogen, USA), and RNA deep sequencing was performed by illumina Novaseq™ 6000. Sequencing results were obtained as FPKM (fragments per kilobase exon per million reads) for each transcript.

### Immunohistochemistry (IHC) and immunofluorescence (IF) staining

IHC and IF staining were performed as previously described by Li et al. [15]. IHC staining was assessed by the immunoreactive score (IRS) method [16].

### Chromatin immunoprecipitation (ChIP)

Predict the binding site sequence of the transcription factor (p-)c-Fos in the target gene MDK promoter region: (1) Use NCBI to obtain the potential promoter region base sequence of the target gene MDK; (2) Use the JASPAR database to query the transcription factor binding site (TFBS) information of c-Fos; (3) Use the JASPAR database to predict the binding site sequence of the transcription factor c-Fos in the target gene promoter region. Total 26 putative sites were predicted with relative profile score threshold 70% (Supplementary Table S3). Then, we performed primer design for the putative site with the highest relative scores (Supplementary Table S4).

The experimental steps are as follows: First, perform formaldehyde crosslinking and sonication of the cells. After sonication, centrifuge at 12,000 rpm, 4 °C for 10 min. Remove the insoluble material and take the supernatant. Take 40 μl of the sonication product as input, add 10 μL of 5* reducing protein loading buffer, heat denatured and perform WB detection to confirm the presence of the target protein in the sample. Take 100 μl and add 900 μl of ChIP Dilution Buffer containing 1mM-PMSF and 20 μl of 50 × PIC (cocktail). Then add 60 μl of Protein A + G Agarose/Salmon Sperm DNA to each tube. Mix well at 4 °C for 1 h. Divide the sample into two 1.5 mL EP tubes, add 1 μg of the target protein IP antibody to one tube, and add 1 μg of corresponding species IgG to the other tube. Mix well at 4 °C overnight. Then perform immunocomplex precipitation and washing, recover DNA samples using a centrifuge column. Regarding the obtained DNA, we initially utilized databases such as JASPAR to predict transcription factor binding sites. Based on these binding sites, primers were designed and synthesized. Subsequently, the accuracy of the fragment size was verified using real-time PCR and agarose gel electrophoresis.

### Co-immunoprecipitation (Co-IP)

First, extract the cell protein, denature it and use it for input experiment, that is, WB detection of the target protein. Then, start the Co-IP experiment: (1) Add 1.0 μg IgG (same species as the IP antibody source) and 20 μL protein A/G beads to the negative control (IgG) group protein supernatant, and directly add 20 μL protein A/G beads to the experimental group. Incubate at 4 °C with shaking for 1 h. (2) After centrifugation, take the supernatant and add 1–10 μL (0.2–2 μg) antibody, then incubate overnight at 4 °C. (3) Add 80 μL protein A/G beads, mix well, and incubate at 4 °C for 2 h. (4) Centrifuge and carefully remove the supernatant, being careful not to suck up the beads at the bottom, and collect the immune precipitation complex. (5) Wash the immune precipitation complex with 1 ml pre-cooled IP lysis buffer, being careful to discard the supernatant after each wash. (6) After the final wash, remove the supernatant as much as possible, then add 80 μL 1× reducing loading buffer, boil for 10 min in boiling water, centrifuge at 4°C and 1000 × *g* for 5 min, and take the supernatant. Label as IP group, and use the prepared protein sample for WB detection of the target protein.

### Intracranial implantation model of glioma

This experiment used 4-6 week old female C57BL/6 mice purchased from GemPharmatech Co., Ltd, with animal qualification certificate number SCXK (Su) 2018-0008. Animal experiments strictly followed the principles of animal in vivo experiment safety and animal experimental ethics approved by Anhui Medical University (LLSC20240354). According to different treatments, they were divided into control group, MDK overexpression group, and MDK overexpression combined with iMDK (MDK inhibitor) group. There were 10 mice in every group.The inhibitor iMDK was injected intraperitoneally (9 mg/kg/day), and the drug was dissolved in DMSO and diluted with a solvent. The dilution scheme was: 10% DMSO + 40% PEG300 + 5% Tween-80 + 45% Saline.

The experimental steps are as follows: first, resuspend 3–5 × 105 GL261 cells infected with lentivirus expressing luciferase in 5 μL serum-free DMEM solution and place on ice. Then, anesthetize the mice with isoflurane and place them on a stereotactic device for fixation. Disinfect the head skin with iodine, fully exposing the central area of the skull, positioning the anterior fontanelle, and drilling the hole 1 mm anterior to the anterior fontanelle and 2 mm to the right of the midline. After positioning, use a micro skull drill to drill the hole, use a micro syringe to extract 5 μL of cell suspension, fix the micro syringe on an automatic micro syringe pump, slowly lower the three-dimensional positioning arm to the needle, move down 4 mm from the drilling hole, then move the needle back 1 mm and start injection. After the injection is complete, leave the needle in the skull for 1 min. Slowly withdraw the needle. After the operation, suture the wound, punch the ear for numbering, and place it on a warming pad. After awakening, put the mouse back in the cage. After the inoculation is completed, use in vivo imaging technology to detect the growth of tumors in the mouse skull every 7 days.

### Flow cytometry

Experimental procedures are shown in the Supplementary Table S5, and the experimental steps are as follows:

For sample A (tumor sample), (1) dissolve the mouse tumor dissociation kit with 3 mL of 1640 or DMEM for each enzyme D, 2.7 mL of 1640 or DMEM for enzyme R, and 1 mL of buffer A for enzyme A. Do not vortex and store at −20 °C. (2) Prepare enzyme MIX: 2.35 mL of 1640 or DMEM + 100 μL of enzyme D + 50 μL of enzyme R + 12.5uL of enzyme A. Each sample is 0.04-1 g, corresponding to 2.5 mL of enzyme mix. (3) After removing the tissue, wash it with PBS to remove fat tissue, fibrous tissue, and necrotic areas. Cut the tumor target area into 2-4 mm pieces and transfer to a C tube containing enzyme mix. (4) Tighten the tube cap and place it on the dissociator. Run the program “m_impTumor_02”. After the program is finished, remove the C tube and place it on a shaker at 37 °C for 40 min. After incubation, run the program according to the tumor texture classification. (5) After the program is finished, remove the C tube, filter all samples through a cell strainer, collect cell counts, and perform post-processing based on the count results, including removing red blood cells, dead cells, and debris until the staining requirements are met.

For sample B (cell surface staining), (1) add an appropriate amount of FC block according to the instructions for each group, incubate in the dark at 2–8 °C for 15 min, then add 1 ml of PBS to wash the cells, centrifuge at 300 × *g* for 5 min, and resuspend in 1 ml. (2) Add an appropriate amount of live/dead dye according to the instructions for each group, incubate in the dark at room temperature for 15 min. Then add 1 ml of stain buffer to wash the cells, centrifuge at 300 × *g* for 5 min, and resuspend in 100 µl. (3) Add an appropriate amount of cell surface fluorescent antibody according to the instructions for each tube, incubate in the dark at 2–8 °C for 30 min. Then add 1 ml of PBS to wash the cells, centrifuge at 300 × *g* for 5 min.

### Statistical analysis

All statistical analyses were performed using R software (versions 3.6.0 and 4.0.2). T-tests and chi-square tests or Fisher’s exact tests were used to compare continuous variables and categorical variables. T-tests were used to analyze differences between two independent groups, and one-way analysis of variance (ANOVA) and least significant difference (LSD) tests were used to analyze differences between more than two groups. GraphPad Prism software was used to analyze the significance differences between Kaplan-Meier survival curves, and Log-rank test was used. A value of *P* < 0.05 was considered statistically significant.

## Results

### Distribution of different *EGFR* mutation forms in GBM patients

Analysis of molecular diagnostic testing data from 71 glioma patients revealed that the incidence of *EGFR* mutations in gliomas was 30.99% (22/71), with *EGFRvIII* mutation being the most common form, accounting for approximately 21.13% (15/71) of all gliomas, followed by EGFR amplification, accounting for approximately 16.90% (12/71) of all gliomas, and finally EGFR point mutation, accounting for approximately 11.27% (8/71) of all gliomas (Fig. S1A, B). In addition, EGFRvIII mutation is associated with glioma WHO grading, molecular subtypes, IDH mutation, and EGFR amplification status. Specifically, compared to EGFRvIII(−) gliomas, the proportion of WHO grade 4 gliomas (EGFRvIII(−):30/56, EGFRvIII(+):14/15, *P* value = 0.044), IDH wildtype GBMs (EGFRvIII(−):24/56, EGFRvIII(+):12/15, *P* value = 0.015), IDH wildtype gliomas (EGFRvIII(−):30/56, EGFRvIII(+):15/15, *P* value = 0.003), and *EGFR*-amplified gliomas (EGFRvIII(−):4/56, EGFRvIII(+):8/15, *P* value < 0.001) was significantly higher in EGFRvIII(+) gliomas (Table 1). These results suggest that gliomas with *EGFRvIII* mutation often indicates higher tumor grades, poorer molecular subtypes, and stronger invasive ability, leading to worse prognosis.

**Table 1:** Comparing molecular and clinical indicators between EGFRvIII(−) (*n* = 56) and EGFRvIII(+) (*n* = 15) GBM patients.

Characteristics	EGFR vIII(−)	EGFRvIII(+)	*P value*
*n*	56	15	
Age (mean (SD))	49.59 (14.61)	55.67 (14.35)	0.155
TMB(mean (SD))	7.75 (21.76)	2.38 (1.98)	0.466
Gender (%)			0.634
Male	36 (64.3%)	8 (53.3%)	
Female	20 (35.7%)	7 (46.7%)	
WHO grade(%)			0.044*
1	1 (1.8%)	0 (0.0%)	
2	15 (26.8%)	1 (6.7%)	
3	10 (17.9%)	0 (0.0%)	
4	30 (53.6%)	14 (93.3%)	
Tumor Type (%)			0.015*
Astrocytoma, IDH-mutant	17 (31.5%)	0 (0.0%)	
Diffuse midline glioma, H3 K27-altered	1 (1.9%)	2 (13.3%)	
Glioblastoma, IDH-wildtype	24(44.4%)	12 (80.0%)	
Oligodendroglioma, IDH-mutant, and 1p/19q-codeleted	8 (14.8%)	0 (0.0%)	
Pilocytic astrocytoma	2 (3.7%)	1 (6.7%)	
Pleomorphic xanthoastrocytoma	2 (3.7%)	0 (0.0%)	
PRS type(%)			0.175
Primary	45 (80.4%)	15 (100.0%)	
Progress	1 (1.8%)	0 (0%)	
Recurrent	10 (17.9%)	0 (0%)	
IDH mutation status (%)			0.003**
Mutant	26 (46.4%)	0 (0.0%)	
Wildtype	30 (53.6)	15 (100.0%)	
1p19q codeletion status(%)			0.231
Codel	9 (16.1%)	0 (0.0%)	
Non-codel	45 (80.4%)	14 (93.3%)	
NA	2 (3.6%)	1 (6.7%)	
MGMTp methylation status(%)			0.412
methylated	26 (46.4%)	4 (26.7%)	
un-methylated	28 (50.0%)	9 (60.0%)	
NA	2 (3.6%)	2 (13.3%)	
EGFR amplification (%)			<0.001***
Yes	4 (7.1%)	8 (53.3%)	
No	48 (85.7%)	4 (26.7%)	
NA	4 (7.1%)	3 (20%)	
EGFR mutation (%)			0.918
no_mutation	46 (82.1%)	9 (60.0%)	
missense_mutation	6 (10.7%)	2 (13.3%)	
NA	4 (7.1%)	4 (26.7%)	
TERT promoter mutation (%)			0.328
Yes	26 (46.4%)	10 (66.7%)	
No	28 (50.0%)	5 (33.3%)	
NA	2 (3.6%)	0 (0.0%)	
+7/−10 copy number changes (%)			0.364
Yes	3 (5.4%)	4 (26.7%)	
No	11 (19.6%)	4 (26.7%)	
NA	42 (75.0%)	7 (46.7%)	
H3_K27M mutation (%)			0.115
Yes	1 (1.8%)	2 (13.3%)	
No	33 (58.9%)	5 (33.3%)	
NA	22 (39.3%)	8 (53.3%)	
CDKN2A/B homozygous deletion (%)			0.821
Yes	5 (8.9%)	4 (26.7%)	
No	11 (19.6%)	5 (33.3%)	
NA	40 (71.4%)	6 (40.0%)	
TP53 mutation (%)			0.107
Yes	24 (42.9%)	2 (13.3%)	
No	30 (53.6%)	11(73.3%)	
NA	2 (3.6%)	2(13.3%)	
ATRX mutation (%)			0.114
Yes	13 (23.2%)	0 (0.0%)	
No	41 (73.2%)	13 (86.7%)	
NA	2 (3.6%)	2 (13.3%)	
BRAF_V600E mutation (%)			1.000
Yes	2 (3.6%)	0 (0.0%)	
No	54 (96.4%)	15 (100.0%)	

*T* tests and chi-square tests or Fisher’s exact tests were used to compare continuous variables and categorical variables. T-tests were used to analyze differences between two independent groups, and one-way analysis of variance (ANOVA) and least significant difference (LSD) tests were used to analyze differences between more than two groups.

*SD* standard deviation, *TMB* tumor mutational burden, *PRS* primary-recurRENt subtype, *IDH* isocitrate dehydrogenase, *MGMTp* O^6^-methylguanine-DNA methyltransferase promoter, *TERT* telomerase reverse tranase, *BRAF* V-raf murine sarcoma viral oncogene homolog B1, *V600E* the most common missense mutation of BRAF, *NA* not available.

*, **, and ***indicate *P* < 0.05, *P* < 0.01, and *P* < 0.001, respectively.

The bolded values signify that their corresponding variables have achieved statistical significance(*P* < 0.05).

### *EGFRvIII* mutation is closely related to immune escape in GBM

**Fig. 1:**
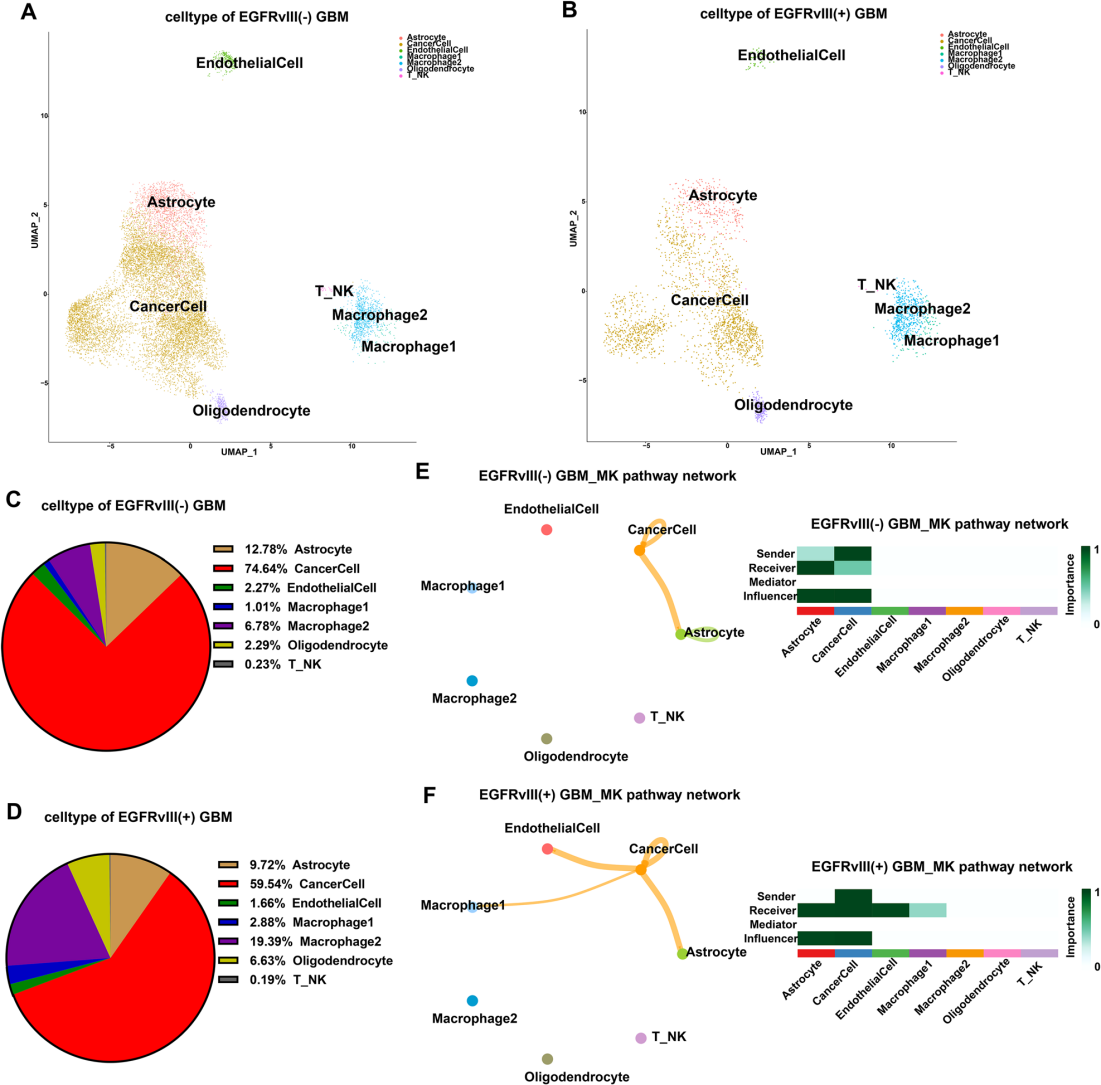
Comparing the tumor microenvironment differences between EGFRvIII(−) and EGFRvIII(+) GBM patients based on single-cell data analysis. **A**, **B** The final UMAP results of the scRNA-seq of EGFRvIII(−) and EGFRvIII(+) GBM patients are also shown. **C**, **D** The immune microenvironment components of EGFRvIII(−) and EGFRvIII(+) GBM patients are presented. **E**, **F** Cell communication analysis was performed based on single-cell data from EGFRvIII(−) and EGFRvIII(+) GBM cells.

We obtained scRNA-seq profiles from six tissue samples, including four EGFRvIII (−) glioblastomas and two EGFRvIII (+) GBMs. Comparative analysis of the immune microenvironment components in EGFRvIII (−) GBMs and EGFRvIII (+) GBMs revealed that cancer cells accounted for 59.54% in EGFRvIII (+) GBMs, followed by M2 macrophages (19.39%), and then astrocytes (9.72%); while cancer cells accounted for 74.64% in EGFRvIII (−) GBMs, followed by astrocytes at 12.78%, and then M2 macrophages at 6.78% (Fig. 1A–D). These data indicate that the largest immune cell subtype in EGFRvIII (+) GBM is M2 macrophages, which are mainly immunosuppressive cells and account for nearly one-fifth of all cell types in EGFRvIII (+) GBM, significantly higher than the M2 macrophage subtype in EGFRvIII (−) GBM, which accounts for only 6.78% of all cell types. This strongly suggests that *EGFRvIII* mutation in GBM is closely related to immune escape.

### The MDK signaling pathway plays an important role between cancer cells and macrophages in GBM with *EGFRvIII* mutation

We performed cell-cell communication analysis on single-cell data from EGFRvIII(−) and EGFRvIII(+) GBM patients separately. CellChat inferred the outgoing and incoming communication patterns of secretory cells. The pattern analysis results for EGFRvIII(−) GBM patient single-cell data (Fig. S2A) revealed that in the outgoing pattern analysis, tumor cells dominated Pattern2, with the MDK signaling pathway being the most active in Pattern2. In the incoming pattern analysis, M1-type macrophages dominated Pattern3, with the ANNEXIN signaling pathway being the most active in Pattern3. For EGFRvIII(+) GBM patient single-cell data (Fig. S2B), the pattern analysis results showed that in the outgoing pattern analysis, tumor cells dominated Pattern3, with the MDK signaling pathway being the most active in Pattern3, followed by the PTN and SPP1 signaling pathways. In the incoming pattern analysis, macrophages dominated Pattern4, with the ANNEXIN signaling pathway being the most active in Pattern4, followed by the GALECTIN and MDK signaling pathways. Further cell-cell interaction analysis of EGFRvIII(+) GBM patient single-cell data indicated no interactions between tumor cells and macrophages in the ANNEXIN and GALECTIN signaling pathways, whereas significant interactions were observed in the MDK signaling pathway (Fig. S2C, D).

**Fig. 2:**
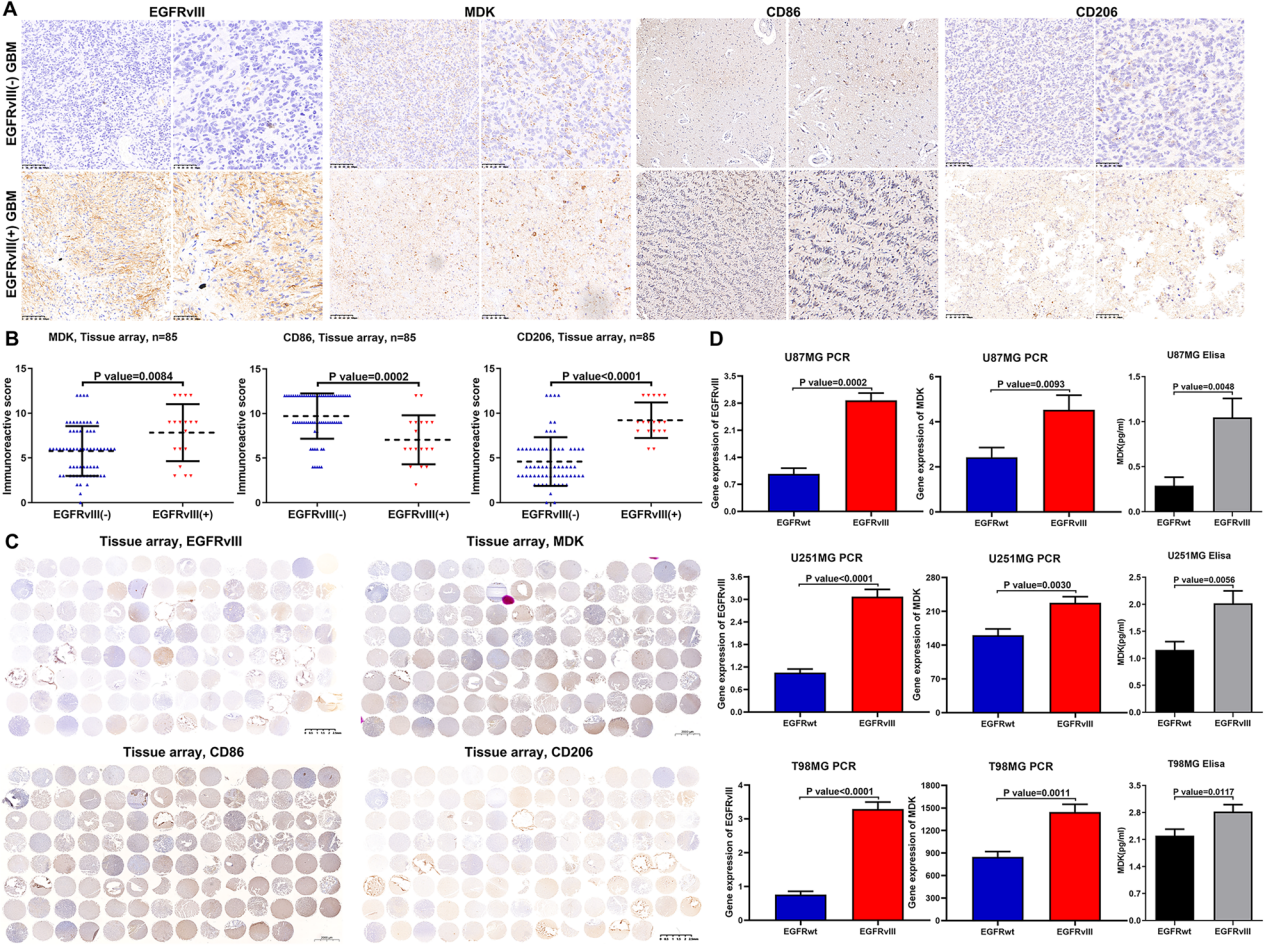
The MDK signaling pathway plays an important role in the interaction between tumor cells and macrophages in EGFRvIII-mutant GBM. **A**–**C** Immunohistochemical staining was performed on tissue microarrays (including EGFRvIII, MDK, CD86 and CD206), and the results were analyzed (Nontumor (*n* = 5), WHO grade 2 (*n* = 30), WHO grade 3 (*n* = 15), WHO grade 4 (*n* = 40)). **D** MDK mRNA and protein expression levels were evaluated by qPCR (*n* = 3) and Elisa (*n* = 3) in human GBM cell lines U87MG, U251MG, and T98MG with wild-type (WT) EGFR and mutant EGFRvIII. unpaired two-tailed Student’s *t* test.

Moreover, we analyzed the communication between cancer cells and immune cells, especially macrophage types, in *EGFRvIII*-mutant GBM to explore the potential mechanism of intercellular interaction and reveal the mechanism of *EGFRvIII* mutation in GBM and immune escape. The results of the cell communication analysis showed that the MDK signaling pathway played an important role in the communication between cancer cells and M1 macrophages in *EGFRvIII*-mutant GBM (Fig. 1E, F). Then, we performed immunohistochemical staining on the tissue microarray using four markers: EGFRvIII, MDK, CD86, and CD206 (Fig. 2C), and selected representative immunohistochemical staining results for these four markers in EGFRvIII(−) and EGFRvIII(+) GBM patients from the tissue microarray (Fig. 2A). Analysis of the tissue microarray staining results revealed that, compared to EGFRvIII(−) GBM patients, the expression of MDK and CD206 was significantly upregulated in EGFRvIII(+) GBM patients, while the expression of CD86 was significantly downregulated in EGFRvIII(+) GBM patients (Fig. 2B). To further validate that *EGFRvIII* mutation contributes to the upregulation of MDK expression, we transfected wild-type (WT) *EGFR* and mutant *EGFRvIII* vectors into human GBM cell lines and evaluated the mRNA and protein expression levels of MDK in U87MG, U251MG, and T98MG cells by qPCR and Elisa. We found that the expression level of MDK mRNA and protein was higher in the *EGFRvIII* mutant group than in the *EGFR* WT group (Fig. 2D). These results suggest that *EGFRvIII* mutation in GBM is likely to regulate macrophages towards an immunosuppressive M2 phenotype through the MDK signaling pathway, ultimately promoting immune escape in GBM.

### Activation of ERK/c-Fos signaling pathway in *EGFRvIII* mutant GBM

**Fig. 3:**
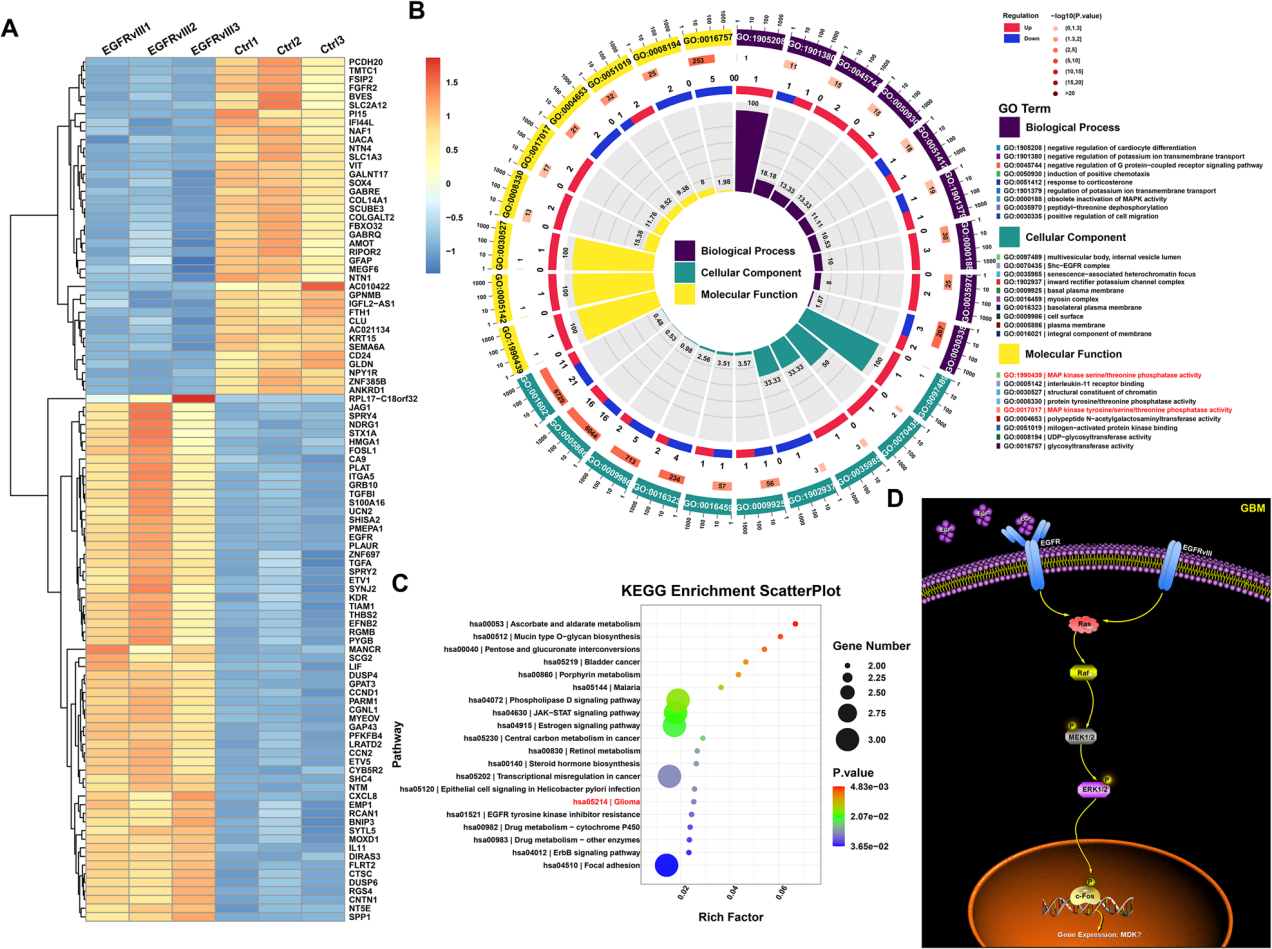
Activation of the ERK/c-Fos signaling pathway in EGFRvIII-mutant GBM. **A** Top 100 DEGs between EGFR wildtype and EGFRvIII-mutant GBM cell lines. **B**, **C** GO and KEGG pathway analysis of differentially expressed genes between EGFR wildtype and EGFRvIII-mutant GBM cell lines. **D** Activation of the MAPK (MEK/ERK/c-Fos) signaling pathway in EGFRvIII-mutant GBM.

To explore how *EGFRvIII* mutant GBM regulates the expression of MDK, we performed transcriptome sequencing on *EGFR* WT and *EGFRvIII* mutant GBM cell lines, and conducted differentially expressed genes (DEGs) analysis on the two groups of samples (fold change >2 or <0.5, and *p* value < 0.05). A total of 106 DEGs were screened, including 54 genes with high expression in the *EGFRvIII* mutant group and 52 genes with low expression in the *EGFRvIII* mutant group (Fig. 3A). Then, transcriptome sequencing results showed that the MAPK signaling pathway was activated in *EGFRvIII* mutant GBM, and ERK/c-Fos, as a key downstream regulatory molecule of this signaling pathway, could directly regulate the expression of downstream targeted genes (Fig. 3B–D).

Furthermore, whether ERK/c-Fos could directly regulate the expression of MDK has become the focus of current research. First, we analyzed the expression distribution characteristics of c-Fos and MDK in GBM pathological grades and molecular subtypes using the TCGA, CGGA, and Rembrandt databases. Compared with normal brain tissue, both c-Fos and MDK were highly expressed in glioma. In accordance with the WHO pathological grading, the expression levels of c-Fos and MDK in glioma increased with the grade (Fig. S3A, E). Similarly, we found that the expression of c-Fos and MDK was highest in IDH wild-type GBM, significantly higher than in other molecular subtypes (Fig. S3B, F). Then, we analyzed the distribution of c-Fos and MDK molecules in different subtypes of primary GBM using the TCGA and CGGA databases. The results showed that compared with neuronal and proneural subtypes of GBM, both c-Fos and MDK were highly expressed in classical and mesenchymal subtypes of GBM (Fig. S3C, G). Further prognostic analysis showed that the prognosis of the high expression c-Fos and MDK group was worse than that of the low expression group in primary glioma patients; similarly, in primary GBM cases, the prognosis of the high expression c-Fos and MDK group was worse than that of the low expression group (Fig. S3D, H). Finally, we analyzed whether there was a correlation between transcription factor c-Fos and MDK in gliomas using TCGA and CGGA databases. The results showed that c-Fos and MDK were correlated in both gliomas and GBMs (Fig. S3I).

To validate the results obtained from the public databases, we performed immunohistochemical staining on the tissue microarray using two markers: c-Fos and MDK (Fig. S4B), and selected representative immunohistochemical staining results for these two markers in normal brain tissue, WHO grade II, WHO grade III, and GBM patients from the tissue microarray (Fig. S4A). Analysis of the tissue microarray staining results showed that compared with the control group, c-Fos and MDK were highly expressed in gliomas; the expression levels of c-Fos and MDK in gliomas increased with the increase in WHO pathological grade; and c-Fos and MDK were significantly correlated in gliomas (Fig. S4C–E). In exploring whether GBMs with *EGFRvIII* mutation could regulate the expression of MDK through the ERK/c-Fos signaling pathway, both public database analysis and tissue microarray analysis suggested that the transcription factor c-Fos was likely to be directly involved in the regulation of MDK expression.

### GBM with *EGFRvIII* mutation could regulate the expression of MDK through the ERK/c-Fos signaling axis

**Fig. 4:**
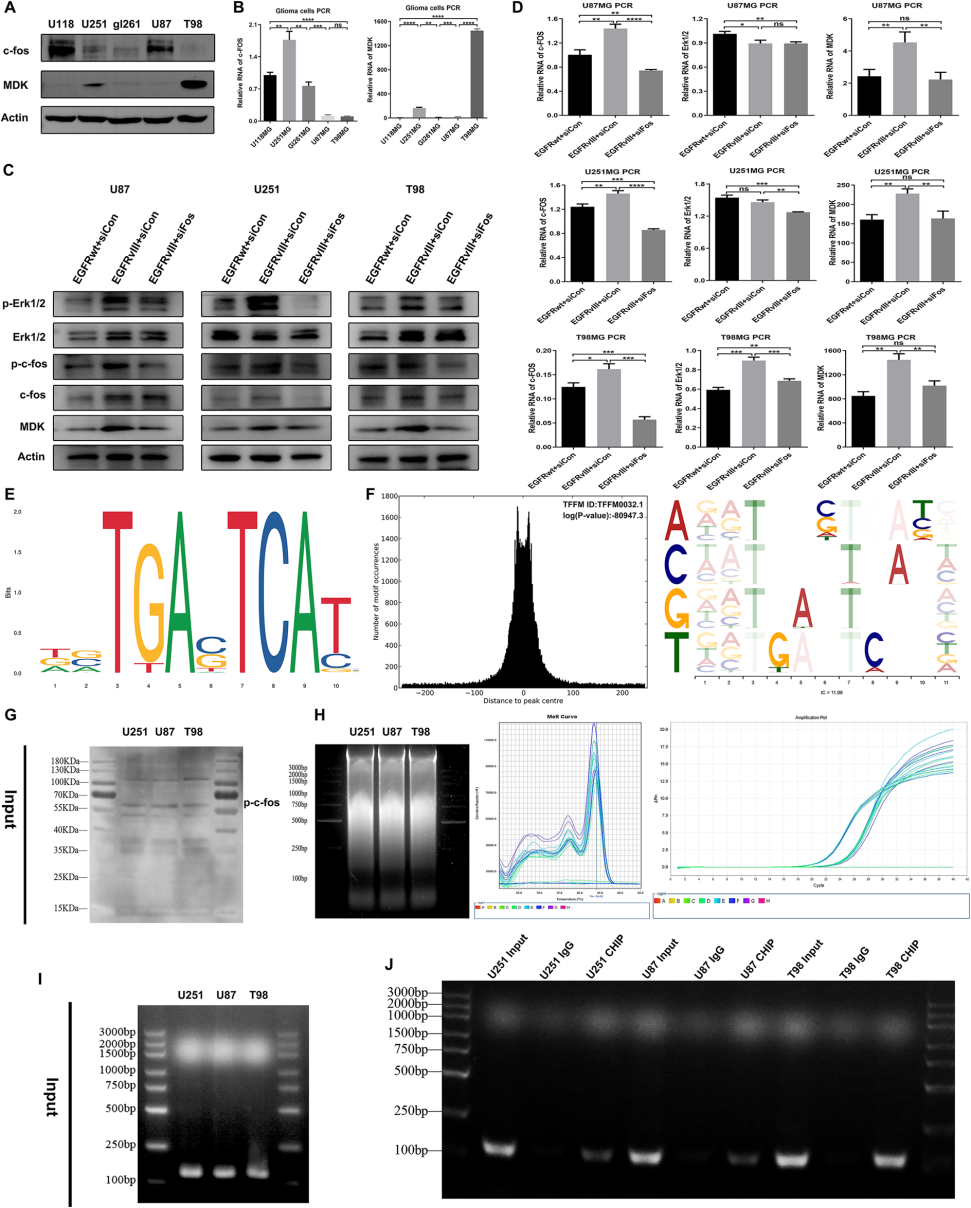
EGFRvIII-mutant GBM regulates the expression of MDK through the ERK/c-Fos signaling axis. **A**, **B** Western blotting (*n* = 3) and qPCR (*n* = 3) were used to detect the expression levels of c-Fos and MDK in common glioma cell lines. **C** Western blotting was used to detect the expression levels of p-Erk1/2, p-c-Fos, Erk1/2, c-Fos, and MDK in different treatment groups of glioma cell lines (*n* = 3). **D** qPCR was used to measure mRNA levels of Erk1/2, c-Fos, and MDK in different treatment groups of glioma cell lines (*n* = 3). **E** Motif logo of transcription factor c-Fos; **F** Centrality plot of ChIP-seq demonstrating the found peak as the transcription factor’s motif location, which is the binding site for the transcription factor; **G** Input western blotting confirmed the presence of the target protein in the sample. **H** The ultrasonic fragmentation of cell chromatin produced chromatin fragments between 100 and 1000 bp in size. The melting curve demonstrates a single peak, and the Tm value of the melting curve is 84.0 °C. The amplification curve displays a region in the exponential growth phase indicating very good reproducibility and a Ct value of 26. **I** Input PCR was used to confirm the correct fragment size. **J** ChIP PCR was used to detect DNA obtained from the precipitation. *, **, *** and **** indicate *P* < 0.05, P < 0.01, *P* < 0.001 and *P* < 0.0001, respectively. Unpaired two-tailed Student’s *t* test.

Through the above transcriptome sequencing analysis, public database analysis, and tissue microarray analysis, we gradually revealed the potential molecular mechanisms of *EGFRvIII* mutation in glioblastomas regulating MDK. The current focus of our research is whether glioblastomas with *EGFRvIII* mutation could directly participate in the regulation of MDK expression through the ERK/c-Fos signaling pathway. First, the expression levels of c-Fos and MDK in common glioma cell lines were detected by Western blotting and qPCR, and based on the results, three cell lines, U87, U251 and T98, were selected for subsequent experiments (Fig. 4A, B). Next, we transfected U87MG, U251MG, and T98MG cell lines with siRNA targeting c-Fos and divided them into three groups: EGFRwt+siCon, EGFRvIII+siCon, and EGFRvIII+siFos. Then, we used Western blotting and qPCR to detect the expression of the ERK/c-Fos signaling pathway and MDK molecule. Western blotting and qPCR results showed that the expression levels of EGFRvIII, p-Erk1/2, p-c-Fos, c-Fos, and MDK were higher in the EGFRvIII+siCon group than in the EGFRwt+siCon group. After adding siRNA targeting c-Fos in the EGFRvIII mutation group, the expression levels of p-Erk1/2, p-c-Fos, c-Fos, and MDK were higher in the EGFRvIII+siCon group than in the EGFRvIII+siFos group (Fig. 4C, D). These results support the view that glioblastoma with *EGFRvIII* mutation promotes MDK expression through the ERK/c-Fos signaling pathway.

To verify whether p-c-Fos can directly regulate MDK expression by binding to its promoter region, we first predicted the binding site sequence of the transcription factor p-c-Fos in the target gene MDK promoter region based on the database. We obtained the potential promoter region base sequence of the target gene MDK using NCBI, and queried the TFBS information of (p-)c-Fos in the JASPAR database (Fig. 4E, F). We then predicted the binding site of p-c-Fos in the target gene MDK promoter region using the JASPAR database (Fig. 4H, Supplementary Tables S3 and S4). ChIP was used to detect whether p-c-Fos can directly bind to the MDK promoter region (TGTGACTCAGT) in U87MG, U251MG, and T98MG cell lines, and the results showed that p-c-Fos can directly bind to the MDK promoter region (Fig. 4G–J). Therefore, the above results indicate that GBM with *EGFRvIII* mutation can directly participate in the regulation of MDK expression through the ERK/c-Fos signaling pathway.

### The MDK secreted by *EGFRvIII* mutant GBM can drive macrophages towards M2 polarization by activating the surface receptor LRP1 and downstream pathways

Cell communication results showed that among all known ligand-receptor pairs, the MDK signal in *EGFRvIII* mutant GBM was mainly controlled by the ligand MDK and its receptor NCL/PTPRZ1/LRP1 (Fig. S5A–D). Further analysis revealed that MDK and NCL/PTPRZ1/LRP1 were highly expressed in cancer cells and astrocytes/cancer cells/macrophages in the MDK signaling pathway, respectively (Fig. S5E, F). Therefore, we hypothesize that the MDK secreted by *EGFRvIII* mutant GBM is likely to drive changes in macrophage phenotype and function by activating the surface receptor LRP1 and downstream pathways.

**Fig. 5:**
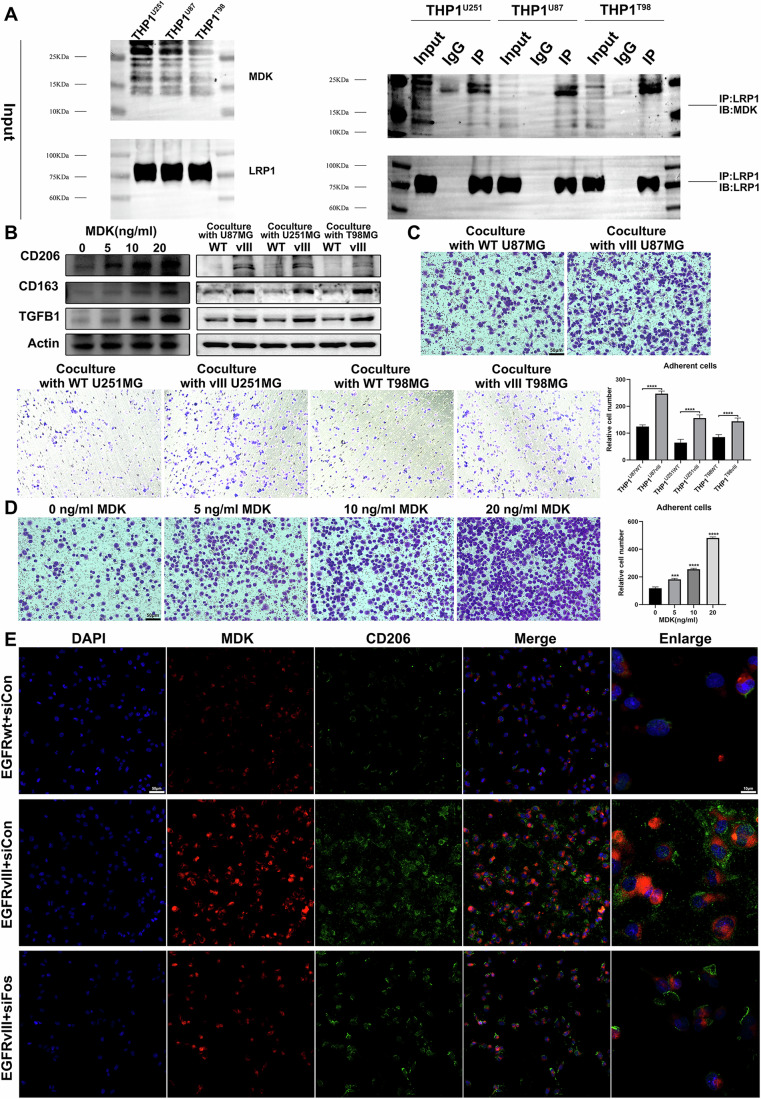
MDK secreted by EGFRvIII-mutant GBM drives macrophage polarization towards M2 through LRP1 receptor activation and downstream pathways. **A** Co-IP experiments were conducted to determine whether the MDK secreted by GBM cells directly binds to the LRP1 receptor on macrophage surfaces. **B** Immunoblotting was used to detect M2 TAMs markers in human monocyte cell line (THP-1) treated with recombinant protein MDK and co-cultured with different glioblastoma cell lines (*n* = 3). **C**, **D** Recombinant protein MDK was used to treat THP-1 cell lines, and transwell co-culture was used to detect whether MDK has a chemotactic effect on macrophages (*n* = 3). **E** Immunofluorescence staining was used to detect the expression and distribution characteristics of MDK and CD206 molecules in THP-1 cells (*n* = 3). *, **, *** and **** indicate *P* < 0.05, *P* < 0.01, *P* < 0.001 and *P* < 0.0001, respectively. Unpaired two-tailed Student’s t test.

To verify whether the MDK secreted by GBM can directly bind to the surface receptor LRP1 on macrophages, we used Co-IP experiment to test THP1^U87MG^, THP1^U251MG^, and THP1^T98MG^ cell lines. The results showed that the MDK secreted by GBM could directly bind to the surface receptor LRP1 on macrophages (Fig. 5A). The protein levels of M2 macrophage markers (CD163, CD206, and TGFB1) were induced after treatment with MDK or co-culture with different EGFRvIII mutant GBM cell lines (Fig. 5B). We further verified whether MDK has a chemotactic effect on macrophages by treating human THP-1 cell line with recombinant MDK protein and co-culturing them with different glioblastoma cell lines. The results showed that the chemotactic effect on macrophages was more obvious when MDK was added to THP-1 or co-cultured with different EGFRvIII mutant GBM cell lines (Fig. 5C, D). Moreover, we used immunofluorescence staining to detect the expression and distribution characteristics of MDK and CD206 molecules in THP-1. The results showed that the expression levels of MDK and CD206 in the EGFRvIII+siCon group were higher than those in the EGFRwt+siCon group. However, after adding siRNA targeting c-Fos to the EGFRvIII group, the expression levels of MDK and CD206 in the EGFRvIII+siCon group were higher than those in the EGFRvIII+siFos group. Fluorescence co-localization analysis showed that both MDK and CD206 were mainly distributed on the cell membrane (Fig. 5E).

Furthermore, we used siRNA targeting MDK to transfect GBM cells and co-cultured them with THP-1. Then, we performed transcriptome sequencing on THP-1 and analyzed the DEGs between the two groups (fold change>2 or <0.5, and p value < 0.05). A total of 204 DEGs were screened, of which 137 were highly expressed in the siMDK co-culture group, while 67 were lowly expressed in the siMDK co-culture group (Fig. S6A). Figure S6B shows the TOP10 DEGs between the siCon and siMDK groups, among which CXCL1, one of the TOP5 DEGs, was significantly downregulated in the siMDK-treated group. Next, enrichment analysis results showed that changes in MDK signaling molecules could affect the functions of signal transduction, immune response, and interaction between cytokines in macrophages (Fig. S6C, D). Finally, the results of immunofluorescence staining showed that the expression levels of MDK, CD206, and CXCL1 in patients with EGFRvIII (+) GBM were higher than those in the EGFRvIII (−) GBM patients. Fluorescence co-localization analysis showed that MDK and CD206 were mainly distributed on the cell membrane (Fig. S6E). We also explored how CXCL1 secreted by macrophages influences the growth of GBM cells through experiments such as CCK-8 assays (Fig. S7B) and Transwell co-culture (Fig. S7A, C–E). The results demonstrated that CXCL1 secreted by macrophages promotes the proliferation and migration of GBM cells.

The above results indicate that MDK secreted by *EGFRvIII*-mutant GBM can activate macrophage surface receptor LRP1 and downstream pathways to drive macrophages towards the immunosuppressive M2 subtype polarization, while promoting macrophage secretion of immunosuppressive cytokine CXCL1 and promoting immune escape of GBM. In turn, CXCL1 secreted by macrophages is capable of promoting the proliferation and migration of GBM cells, ultimately leading to malignant progression of GBM.

### Targeted regulation of the MDK signaling pathway in GBM can affect tumor growth and the immune microenvironment

**Fig. 6:**
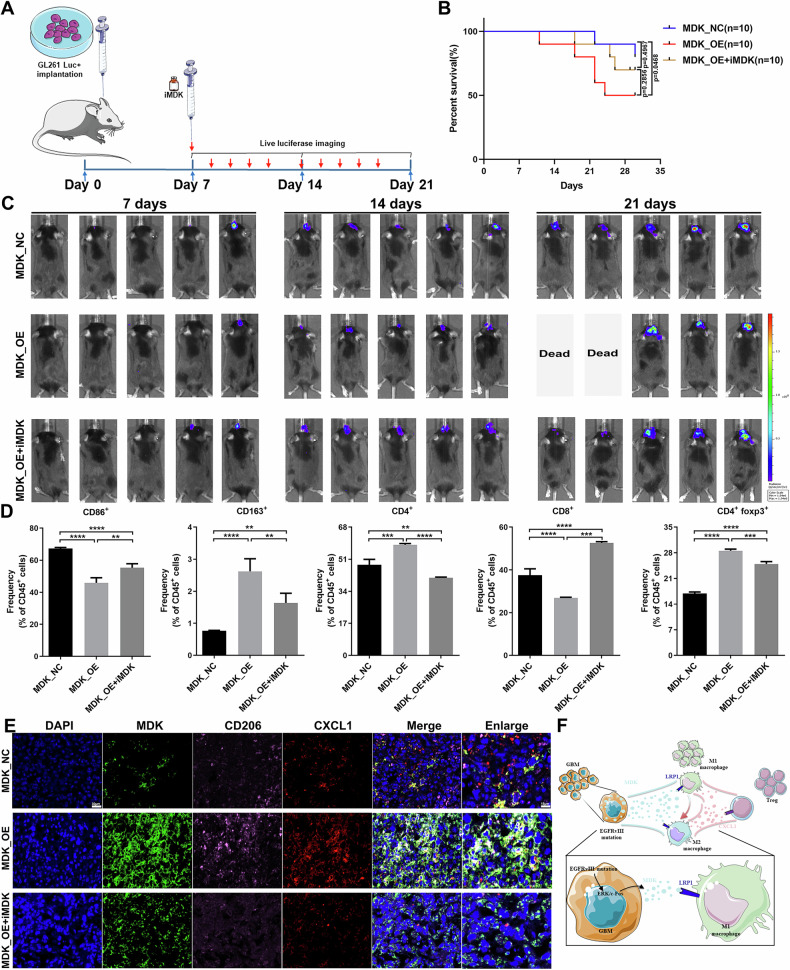
Targeting the MDK signaling pathway in GBM can affect intracranial tumor growth and immune microenvironment. **A** A mouse GBM intracranial implantation model was established by implanting stable overexpression of MDK molecule GL261 cell lines into the right striatum of mice, divided into MDK_NC, MDK_OE, and MDK_OE+iMDK (*n* = 10) according to different treatment methods. **B**, **C** In vivo imaging technology was used to monitor intracranial tumor growth every week, and the Kaplan–Meier survival curve was used to analyze the differences between the groups (*n* = 10). **D** Flow cytometry was used to detect immune cell subpopulations (M1 and M2 macrophages, Tregs, CD4+ and CD8+ T cells) in the brain tumor immune microenvironment of control group, MDK overexpression group, and MDK overexpression combined with iMDK treatment group (*n* = 3). **E** Multiple immunofluorescence staining was used to detect the expression and distribution characteristics of MDK, CD206, and CXCL1 molecules in control group, MDK overexpression group, and MDK overexpression combined with iMDK treatment group (*n* = 3). **F** Molecular mechanisms diagram of immune evasion in GBM with EGFRvIII mutation. *, ** and **** indicate *P* < 0.05, *P* < 0.01 and *P* < 0.0001, respectively. unpaired two-tailed Student’s *t* test. The overall survival of mice was analyzed for prognosis using the Kaplan-Meier method.

To further investigate the effect of targeted regulation of the MDK signaling pathway in glioblastoma on the growth and prognosis of intracranial tumors, we established a mouse glioma intracranial model. GL261 cells stably overexpressing MDK were implanted in the right striatum of mice (Fig. 6A) and divided into control group, MDK overexpression group, and MDK overexpression plus iMDK combined application group according to different treatment methods. The results showed that compared with the control group, the volume of GBM in the MDK overexpression group was larger, while the tumor growth in the MDK overexpression plus iMDK combined application group was slow (Figs. 6C and S8), which was also supported by HE staining results (Fig. S9). In addition, we also counted the survival status of mice. Compared with the control group, the survival period of mice in the MDK overexpression group was shorter, while the combined application of MDK overexpression plus iMDK significantly prolonged the survival period of mice (Fig. 6B).

To explore the effect of targeted regulation of the MDK signaling pathway in GBM on the immune microenvironment within the tumor, we also used immunohistochemical staining technology to detect the expression characteristics of MDK, CXCL1, Ki67, CD3, CD4, CD8, CD68, iNOS, CD206, PD1, PD-L1, and Foxp3 molecules. The results of immunohistochemical staining showed that the expression levels of CXCL1, Ki67, CD4, and CD206 in the MDK overexpression group were higher than those in the control group, while the expression levels of MDK, CXCL1, Ki67, CD4, and CD206 in the MDK overexpression plus iMDK combined application group were downregulated compared with the MDK overexpression group (Fig. S9). Then, we used flow cytometry to detect the immune cell subpopulations (M1 and M2 type macrophages, Tregs, CD4+ and CD8+ T cells). The results showed that the proportion of M2 type macrophages, Tregs and CD4+ T cells in the MDK overexpression group was significantly higher than that in the control group, while the proportion of these cells was reduced in the MDK overexpression plus iMDK combined application group compared to the MDK overexpression group (Figs. 6D, and S10). Finally, we also used immunofluorescence staining to detect the expression and distribution characteristics of MDK, CD206, and CXCL1 molecules. The results showed that the expression levels of MDK, CD206, and CXCL1 in the MDK overexpression group were higher than those in the control group, while the expression levels of these molecules were downregulated in the MDK overexpression plus iMDK combined application group compared to the MDK overexpression group. Fluorescence co-localization analysis showed that MDK and CD206 were mainly distributed on the cell membrane, while CXCL1 was mainly distributed outside the cell (Fig. 6E). These results indicate that targeting the MDK signaling pathway can inhibit the polarization of macrophages towards the immune suppressive M2 subtype and the secretion of the immune suppressive cytokine CXCL1, reverse the formation of the immune suppressive microenvironment, and slow down the malignant progression of GBM (Fig. 6F).

## Discussion

Genomic alterations can reshape the TME and drive the malignant progression of GBM [17]. The interaction between tumor cells and macrophages plays a crucial role in tumor development, supporting angiogenesis, nurturing cancer stem cells, and promoting the immune suppressive TME [18, 19]. TME is composed of cellular and molecular components that support tumor progression [20]. In addition to highly heterogeneous tumor cells themselves, the TME of glioblastoma also includes many different non-cancerous cell types, including endothelial cells, pericytes, fibroblasts, and immune cells [21]. The majority of immune cells in GBM are TAMs, which can account for up to 40% of the tumor mass [22]. TAMs can weaken anti-tumor immunity and promote the malignant behavior of glioblastoma, facilitating tumor growth. The immunosuppressive macrophage subtype M2 TAMs not only promote angiogenesis, but also facilitate invasion, migration, and intravasation of tumor cells. It also serves as an immune suppressor, preventing tumor cells from being attacked by natural killer cells and T lymphocytes during the application of immunotherapy in cancer treatment [23]. Various therapeutic approaches can target TAMs, including blocking the recruitment of peripheral macrophages, directly consuming TAMs, and re-educating TAMs from an immunosuppressive subtype (M2 TAMs) to an anti-tumor phenotype (M1 TAMs), all of which aim to reduce the number of M2 TAMs in the TME. The goal of these therapies is to reshape the tumor immune microenvironment, reduce immune suppression, and provide effective ways to inhibit tumor progression [24]. However, in the research process, some researchers have found that targeting the colony-stimulating factor 1 receptor (CSF-1R) to reduce macrophage polarization towards M2 and block GBM malignant progression resulted in acquired resistance in animal models [25], and the efficacy was found to be very limited in clinical trials [26]. Therefore, it is crucial to identify other strategies to improve the effectiveness of targeted therapy for macrophages in GBM patients.

This study for the first time found that the largest immune cell subset in EGFRvIII (+) GBM is the M2 macrophage subtype, with M2 macrophages accounting for nearly one-fifth of all cell types in EGFRvIII (+) GBM, significantly higher than the M2 macrophage subset in EGFRvIII (−) GBM (only accounting for 6.78% of all cell types). Moreover, intercellular communication analysis revealed that the MDK signaling pathway plays an important role in the communication between cancer cells and macrophages in *EGFRvIII*-mutant GBM. Tissue microarray, immunofluorescence, and qPCR experiments confirmed that the M2 TAMs marker CD206 and the key molecular MDK expressed mainly in cancer cells are highly expressed in *EGFRvIII*-mutant GBM, suggesting that the MDK molecule is involved in the regulation of cancer cells on surrounding TAMs in *EGFRvIII*-mutant GBM.

The study found that midkine (MDK) is not only abnormally highly expressed in various cancers, but as a secreted protein, MDK could activate downstream signaling cascade reactions through interaction with receptors or receptor complexes, and these downstream signaling events may be related to a variety of phenotypic features that lead to cancer development, including cancer cell growth, migration, metastasis, and angiogenesis [27, 28]. MDK expression in cancers can also be detected early, making it a relevant biomarker for cancer progression. Furthermore, its expression in tumors can be detected through blood and urine analysis [29]. MDK is also one of the growth factors that regulate inflammation and has specific functions in MDK-mediated inflammation [30]. MDK could support the adhesion of polymorphonuclear neutrophils (PMNs) by promoting high-affinity β2-integrin, thereby promoting the transport of PMNs during the acute inflammatory phase. Inhibiting/blocking low-density lipoprotein receptor-related protein 1 (LRP1) affects this process, indicating that it may be an MDK receptor on PMNs [31]. In addition to neutrophils, MDK could also regulate the chemotaxis of macrophages. MDK-deficient mice showed lower numbers of neutrophils and macrophages in a model of early fracture healing [32]. To date, there have been few studies on the immune regulatory functions and potential mechanisms of MDK in the TME [33]. Relevant studies have found that using PBMCs from healthy donors can induce human CD8 + T cells to respond to MDK-expressing cancer cells in vitro; and due to its widespread expression in cancer tissue and its contribution to cancer development, MDK appears to be an attractive candidate for cancer vaccines [34]. Given the limited research mentioned above, the functions and mechanisms of MDK in neutrophil and macrophage-mediated cancer inflammation still need further experimental verification and clarification.

Therefore, in order to clarify the impact and potential mechanisms of *EGFRvIII* mutation on the immune microenvironment in GBM, through a series of molecular biology experiments, it was demonstrated that *EGFRvIII*-mutated GBM is directly involved in the regulation of MDK expression through the ERK/c-Fos signaling pathway. Meanwhile, MDK secreted by *EGFRvIII*-mutated GBM could activate macrophage surface receptor LRP1 and downstream pathways to drive macrophages towards the immunosuppressive phenotype M2 type polarization, promoting the formation of an immunosuppressive TME.

Chemokine (C-X-C motif) ligand 1 (CXCL1) is a small-molecule cytokine belonging to the CXC chemokine family [35]. Recent research evidence shows that the high expression level of CXCL1 in different types of cancers is associated with advanced cancer stage, larger cancer volume, cancer invasiveness, and poor prognosis. CXCL1 is commonly overexpressed in cancer and stromal cells of various cancers, including breast cancer, colorectal cancer, prostate cancer, pancreatic ductal adenocarcinoma, and bladder cancer [36]. CXCR2, a G protein-coupled chemokine receptor, specifically binds to CXCL1 and is mainly expressed on neutrophils and macrophages derived from bone marrow, to recruit MDSCs and TAMs [37]. There is ample evidence that MDSCs and TAMs in cancers suppress the expansion and effector function of T cells by inducing checkpoint regulators and exhaustion markers, thereby protecting the survival and metastasis of cancer cells. Conversely, the infiltration of MDSCs and TAMs may recruit Treg cells to promote highly immunosuppressive TME in these cancers [38]. Studies have shown that CXCL1 is overexpressed in GBM and closely related to its invasiveness. Blocking CXCL1 reduces the migration of MDSCs, TAMs, and Tregs to the tumor, thus significantly alleviates the highly immunosuppressive microenvironment of GBM and leads to the high aggregation of CD8+ T cells, thereby reactivating the anti-tumor immune response against GBM [39].

We further explored the effects of MDK secreted by EGFRvIII-mutated GBM cells on macrophage function and in turn how macrophages influence the growth of GBM cells. The results indicate that MDK secreted by *EGFRvIII*-mutated GBM can promote the secretion of the immunosuppressive cytokine CXCL1 by macrophages, promoting the formation of an immunosuppressive microenvironment. In turn, CXCL1 secreted by macrophages is capable of promoting the proliferation and migration of GBM cells, ultimately leading to malignant progression of GBM.

iMDK (MDK inhibitor, molecular weight 376.4) was first reported to inhibit MDK protein levels through screening. In vitro, iMDK inhibited the growth of lung cancer cells by inhibiting MDK protein levels. In addition, intravenous injection of iMDK inhibited tumor growth in a nude mouse model of lung cancer [40]. iMDK also inhibited the growth and angiogenesis of oral squamous cell carcinoma [41]. Treatment with IFN-γ activates the epithelial-mesenchymal transition (EMT) program and the metastasis of various cancer cells. MDK is overexpressed in most cancer types and its overexpression drives the activation and metastasis of cancer cells through EMT. Targeted inhibition of MDK using iMDK can broadly reverse IFN-γ-activated EMT and eliminate the metastasis of various cancers caused by IFN-γ. The combined use of MDK inhibitors can significantly enhance the anti-tumor activity of IFN-γ [42, 43].

As an effector molecule downstream of the ERK/c-Fos signaling pathway in *EGFRvIII*-mutated GBM, MDK suggests a potential therapeutic target for this subtype of GBM. This study explored treatment strategies targeting *EGFRvIII*-mutated GBM and investigated the effects of targeted regulation of the MDK signaling pathway on tumor growth, prognosis, and the immune microenvironment in a mouse model of intracranial glioma. The results of animal experiments indicate that targeted blockade of the MDK signaling pathway can reverse the formation of an immunosuppressive microenvironment, slow the malignant progression of gliomas, and prolong the prognosis of mice.

## Conclusion

In summary, this study first discovered that *EGFRvIII*- positive GBM could drive macrophages towards M2 polarization and secrete the immunosuppressive cytokine CXCL1 by activating the c-Fos-MDK-LRP1 signaling pathway, providing a new target for precise immunotherapy for specific subtypes or genotypes of GBM. Secondly, since immune therapies such as immune checkpoint inhibitors have not been successful in phase III clinical trials for newly diagnosed or recurrent glioblastoma, the combination of immune therapies and targeted EGFRvIII or downstream pathway molecules provides a molecular basis and new ideas for the current dilemma of glioma immunotherapy.

## Supplementary information

Supplementary Information

Supplementary Table S1

Supplementary Table S2

Supplementary Table S3

Supplementary Table S4

Supplementary Table S5

Original Western Blots

sFigure 1

sFigure 2

sFigure 3

sFigure 4

sFigure 5

sFigure 6

sFigure 7

sFigure 8

sFigure 9

sFigure 10

## Data Availability

The data analyzed in this study were obtained from the University of California Santa Cruz Cancer Genomics Browser (https://genome-cancer.ucsc.edu), and from The Cancer Genome Atlas database (https://tcga-data.nci.nih.gov/docs/publications/lgggbm_2015/), and from the Chinese Glioma Collaborative Group website (http://www.cgga.org.cn/), and from the National Cancer Institute Molecular Brain Tumor Data Storage Repository (https://gdoc.georgetown.edu/gdoc) queue, and from Gene Expression Omnibus (http://www.ncbi.nlm.nih.gov/geo/) at GSE141383. Code used in the current research are available from the corresponding author upon reasonable request.
