# Supplementary material for: EGFRvIII-positive glioblastoma contributes to immune escape and malignant progression via the c-Fos-MDK-LRP1 axis
Source: Cell Death Dis. 2025 Jun 17;16(1):453. doi: 10.1038/s41419-025-07771-1 (PMC12174314; doi:10.1038/s41419-025-07771-1)
Supplement: Supplementary file 1 — Supplementary Information [file 41419_2025_7771_MOESM1_ESM.docx]

**EGFRvIII-positive glioblastoma contributes to immune escape and malignant progression via the c-Fos-MDK-LRP1 axis**

**Supplementary Materials**

**1. Antibody information list**

| **Antibody** | **Production company** | **Product number** |
| --- | --- | --- |
| EGF Receptor vIII (D6T2Q) XP® Rabbit mAb | CST | 64952T |
| Anti-Midkine [EP1143Y] | Abcam | ab52637 |
| CD206 Mouse Polyclonal Antibody | Proteintech | 60143-1-Ig |
| Midkine (MDK) Mouse Monoclonal Antibody [Clone ID: OTI8C6] | OriGene | TA807775 |
| Anti-LRP1 [EPR3724] | Abcam | ab92544 |
| c-Fos (9F6) Rabbit mAb | CST | 2250T |
| c-Fos (E7L5L) Mouse mAb | CST | 74620S |
| Anti-c-Fos (phospho T325) | Abcam | ab27793 |
| Anti- c-Fos Rabbit pAb | Servicebio | GB114125 |
| c-Fos (6-2H-2F) | Santa | sc-447 |
| p44/42 MAPK (Erk1/2) Antibody | CST | 9102S |
| Phospho-p44/42 MAPK (Erk1/2) (Thr202/Tyr204) Antibody | CST | 9101S |
| Anti -Phospho-ERK1 (T202/Y204) + ERK2 (T185/Y187) Rabbit pAb | Servicebio | GB11004 |
| human mldkine | Absin | abs00930 |
| CXCL1 Polyclonal antibody | PTG | 12335-1-AP |
| Anti -beta Actin Rabbit pAb | Servicebio | GB11001 |
| CD3ε (E4T1B) XP® Rabbit mAb | CST | 78588T |
| CD4 (D7D2Z) Rabbit mAb | CST | 25229T |
| CD8α (D4W2Z) XP Rabbit mAb (Mouse Specific) | CST | 98941T |
| PA-ANTI-MO/RT FOXP3 FJK-16S PUR | Invitrogen | 14-5773-80 |
| CD68 (E3O7V) Rabbit mAb | CST | 28058-1-AP |
| INOS Polyclonal Antibody | Proteintech | 22226-1-AP |
| CD163 Rabbit Polyclonal Antibody | Proteintech | 16646-1-AP |
| PD-L1/CD274 Mouse Monoclonal Antibody | Proteintech | 66248-1-Ig |
| Ki-67 Polyclonal Antibody | ImmunoWay | YT2467 |
| ANTI-MO CD3 17A2 FITC | eBioscience | 11-0032-80 |
| ANTI-MO CD4 RM4-5 PERCP-CYN5.5 | eBioscience | 45-0042-80 |
| ANTI-MO CD8A 53-6.7 APC | eBioscience | 17-0081-81 |
| ANTI-M/R FOXP3 FJK-16S PE | eBioscience | 12-5773-82 |
| FIXABLE VIABILITY DYE EF450 | eBioscience | 65-0863-14 |
| RAT IGG2BK ISOCTRL FITC | eBioscience | 11-4031-81 |
| RAT IGG2AK ISO CTRL EBR2A PE | eBioscience | 12-4321-80 |
| RT IGG2AK ISO CTL PERCP-CYN5.5 | eBioscience | 45-4321-80 |
| RAT IGG2AK ISO CTRL EBR2A APC | eBioscience | 17-4321-81 |
| ANTI-MO CD163 TNKUPJ APC | eBioscience | 17-1631-80 |
| ANTI-MO CD86 GL1 PE | eBioscience | 12-0862-81 |
| ANTI-MO CD11B M1/70 FITC | eBioscience | 11-0112-81 |
| FOXP3/TRN FACTOR STAIN BUFFER | eBioscience | 00-5523-00 |
| APC/Fire™ 750 anti-mouse CD45 | BioLegend | 103153 |
| ANTI-M F4/80 AG BM8 PCP-CYN5.5 | eBioscience | 45-4801-80 |
| ULTRACOMP PLUS BEADS, 100 TESTS | eBioscience | 01-3333-42 |
| Ms CD45 BV510 30-F11 50ug | BD Pharmingen | 563891 |

**2. The primer sequences for qPCR**

| **Primer** | **Sequence（5'-3'）** |
| --- | --- |
| human-EGFRvIII-F | GTGGATGCCGACGAGTACCTC |
| human-EGFRvIII-R | ACAGCTTTGCAGCCCATTTCTAT |
| Human-MDK-F | AAGGATTGCGGCGTGGGTTTC |
| Human-MDK-R | TGGCGGACTTTGGTGCCTGTG |
| human-c-Fos-F | CTCCAGTGCCAACTTCATTCCC |
| human-c-Fos-R | GCCATCTTATTCCTTTCCCTTCG |
| mouse -FOS-F | ACCCTTTGATGACTTCTTGTTTCC |
| mouse -FOS-R | TTGCTGTGCAGAGGCTCCC |
| mouse -MDK-F | GCCGACTGCAAATACAAGTTTGAGA |
| mouse-MDK-R | GGGCTTAGTCACGCGGATGGT |
| human-ERK1-F | AAAGCCAGCAGCTGAGCAAT |
| human-ERK1-R  human-CXCL1-F  human-CXCL1-R | TTAAGGTCGCAGGTGGTGTT  AGCTTGCCTCAATCCTGCATCC  TCCTTCAGGAACAGCCACCAGT |
| Human-GAPDH-F | AATCCCATCACCATCTTCCA |
| Human-GAPDH-R | AAATGAGCCCCAGCCTTCT |

**3. The sequences of siRNAs used for gene silencing**

| **siRNAs** | **Target RNA Sequence** |
| --- | --- |
| siCon | UGGUUUACAUGUCGACUAA |
| siFOS | GCGAGGAGACAGCCAAUUA |
| siCXCL1 | CCAAGAACAUCCAAAGUGUTT |

**4. (p-)c-Fos promoter region binding site prediction and primer design.**

cFOS→MDK，Human

>NC_000011.10:46378784-46380784 Homo sapiens chromosome 11, GRCh38.p14 Primary Assembly

GCTGCCAGAGAGCCCACAGGCTGTGGGGTGAGAGGCCCCTCCTCCGGTGTGCTCCAGAGAGACCCACAGAGGCAACTCAGGAGTAAGATGTGTGAGCGCACTCGTTTCAGGCCTGTGCTGGTGTACCCGGCTGTGGGCCAGCGTGTGAGCTCAGGGAAGGAGGGGTGGCCCCAGGAGGTCCAGCCCTGCCATGCCTCCTGCCCTCAGCTCCAGGAGCTGCACCGAGCTGGGGGCGACCTCATGCACCGAGACGAGCAGAGTCGCACGCTCCTGCACCACGCAGTCAGCACTGGCAGCAAGGATGTGGTCCGCTACCTGCTGGACCACGGTGAGCCGGGCAGTGGAGCCCAGGGCCTGGGGCCAAGGTGGGAGGAGGAGGGGCTGCCAGGCCTCTCTGACCACCACCTCCCCTTCTCCAGCCCCCCCAGAGATCCTTGATGCGGTGGAGGAAAAGTAAGTATCTGGGCAGTGCAGAACCGTGGTCACCCCGGAAACCACCCTTTTCCCCACCCCTCCCATTTTGTCAGGTCAGAGCCCATAAACTTCCTGGTCACATCTGTCATCCCCTGGGCCACCCCTATTGCCCCAGAGCCCTGAACTTCCTGCCCTTTCTGATGGCCCTTGGGAGACAGATGGGTGGATCAGGGGACGGGATGGGGTACACAGCCAGCCCCTGCTCCCCCAGCGGGGAGACCTGTTTGCACCAAGCAGCGGCCCTGGGCCAGCGCACCATCTGCCACTACATCGTGGAGGCCGGGGCCTCGCTCATGAAGACAGACCAGCAGGTGAGCAGACGGCAGGCAGGGAGCCCACGAGGGCACCAACCAAACCTTTCCCAAGGTCCTAGGCGGGAGCTGGGGCTGGGGGCTGTCCCTGGGAAGACACAGTCCAGACCCTGGGAAACCTGAGCCAGCAGGGGAGGAGCTGGTGGGCAGAGAGGCCTCCCTCCCTGACCAGGCCACAGGGAGGTAGAGCCCCTGCCTCTCAGCCTGCTAGGGGTTAGGCCTGCCTCTGGCCCCTGCTGATCGCAGCTCCGCCCTCCTCCAGGGCGACACTCCCCGGCAGCGGGCTGAGAAGGCTCAGGACACCGAGCTGGCCGCCTACCTGGAGAACCGGCAGCACTACCAGATGATCCAGCGGGAGGACCAGGAGACGGCTGTGTAGCGGGCCGCCCACGGGCAGCAGGAGGGACAATGCGGCCAGGGGACGAGCGCCTTCCTTGCCCACCTCACTGCCACATTCCAGTGGGACGGCCACGGGGGGACCTAGGCCCCAGGGAAAGAGCCCCATGCCGCCCCCTAAGGAGCCGCCCAGACCTAGGGCTGGACTCAGGAGCTGGGGGGGCCTCACCTGTTCCCCTGAGGACCCCGCCGGACCCGGAGGCTCACAGGGAACAAGACACGGCTGGGTTGGATATGCCTTTGCCGGGGTTCTGGGGCAGGGCGCTCCCTGGCCGCAGCAGATGCCCTCCCAGGAGTGGAGGGGCTGGAGAGGGGGAGGCCTTCGGGAAGAGGCTTCCTGGGCCCCCTGGTCTTCGGCCGGGTCCCCAGCCCCCGCTCCTGCCCCACCCCACCTCCTCCGGGCTTCCTCCCGGAAACTCAGCGCCTGCTGCACTTGCCTGCCCTGCCTTGCTTGGCACCCGCTCCGGCGACCCTCCCCGCTCCCCTGTCATTTCATCGCGGACTGTGCGGCCTGGGGGTGGGGGGCGGGACTCTCACGGTGACATGTTTACAGCTGGGTGTGACTCAGT (Binding site) AAAGTGGATTTTTTTTTCTTTTC TGCTTTTCTTCTTTTGCGGGGGAGGTCTAACAAGCAGCGGGGGCTGCGGGGTTGTCCTCGGGGTGGGGGACTGGACGCTGTCGACAGCACCTTCCTGGGGCCCCGGCTCCCGTTTGGTGGTTGGTCCCAGGGCCTGCCCGGTTCCTGACCTCTGCCCGGCGGCCGCGCTCGTCGGGGCCGGGGGCGGGGCCGATCCCTCCGGCTTCCCGCTTCCCGCGGAGAACAACA

| **Name** | | **Primer sequence (5'to3')** | **Fragment size (bp)** |
| --- | --- | --- | --- |
| Chip-RT-PCR | F | GCTCCCCTGTCATTTCATCG | 133 |
|  | R | CCGCAAAAGAAGAAAAGCAGAA |  |

**5. Flow cytometry antibody list and experimental protocol for flow cytometry.**

| **Subtype** | **Indicator** | **Fluorescein** | **Product number** |
| --- | --- | --- | --- |
| Panel 1 | CD45 | BV510 | 563891 |
|  | CD11B | FITC | 11-0112-81 |
|  | CD86 | PE | 12-0862-81 |
|  | CD163 | APC | 17-1631-80 |
|  | Live/Dead | APC-CY7 | 565388 |
| Panel 2 | CD45 | BV510 | 563891 |
|  | CD3 | FITC | 11-0032-80 |
|  | CD4 | PERCP-CY5.5 | 45-0042-80 |
|  | CD8 | APC | 17-0081-81 |
|  | FOXP3 | PE | 12-5773-82 |
|  | Live/Dead | APC-CY7 | 565388 |

**Supplementary Methods**

**1. Cell lines and cell culture**

Human glioma cell lines U87MG, U251MG, T98MG, and mouse glioma cell line GL261 were purchased from the Cell Resource Center of the Shanghai Institute of Life Sciences, Chinese Academy of Sciences. Glioma cells were cultured in high-glucose DMEM medium containing 10% heat-inactivated fetal bovine serum (FBS), 100 U/ml penicillin, and 0.1 mg/ml streptomycin and incubated in a constant temperature incubator at 37°C and 5% CO2 saturation. Human THP-1 (human monocytic leukemia) cell line was kindly provided by Professor Zhang Junxia's team from the First Affiliated Hospital of Nanjing Medical University. Human THP-1 cells were cultured in RPMI-1640 medium containing 10% heat-inactivated FBS, 100U/ml penicillin, and 0.1mg/ml streptomycin.

**2. Co-culture of cells**

Human glioma cells and THP-1 cells were co-cultured using Transwell chamber. The Transwell chamber was placed in a six-well plate, with a layer of PC or PET membrane between the upper and lower chambers. The membrane had micropores with a maximum diameter of 12.0um and a minimum diameter of 0.1um. When the pore size was less than 3.0um, cells could not pass through the membrane, but cytokines and other molecules could freely pass through. The steps for co-culture were as follows: (1) THP-1 cells were induced to differentiate into macrophages with 185ng/ml Phorbol-12-myristate-13-acetate (PMA); (2) Cells were digested, centrifuged, and finally resuspended; (3) Human glioma cells and PMA-induced THP-1 cells were separately seeded in the upper and lower chambers of the Transwell, with a 1:1 ratio of cells in the upper chamber with high-glucose DMEM as the culture medium and lower chamber with RPMI-1640 as the culture medium. (4) After co-culture, the cell status in the upper and lower chambers was observed under a microscope. If necessary, relevant staining experiments could be performed. Cells in the upper and lower chambers could also be collected for RNA or protein extraction to detect target genes, as well as other phenotype experiments (generally after 48-72 hours of treatment).

**3. Single-cell RNA-seq analysis**

We obtained single-cell RNA sequencing (scRNA-seq) profiles from 16,028 single cells derived from six tissue samples, including four EGFRvIII(-) glioblastoma cases and two EGFRvIII(+) glioblastoma cases. After applying quality control criteria, 14,013 single cells were retained for further analysis. To visualize the distribution of the scRNA-seq profiles, we applied t-distributed Stochastic Neighbor Embedding (t-SNE) to reduce the dimensionality of these datasets. Batch effects within these datasets were appropriately corrected using the "fastMNN" algorithm. Through unsupervised clustering, the cells were successfully grouped into 17 clusters. Based on the expression patterns of markers from the CellMarker database, we manually annotated these clusters into the following seven cell types: 1) GBM cancer cells (expressing SOX2, PARP1, and CCND2); 2) M1-type macrophages (expressing CD68, CD74, TSPO, and CD86); 3) M2-type macrophages (expressing CD68, CD74, and CD163); 4) T cells or NK cells (expressing CXCR4 and S100A4); 5) endothelial cells (expressing A2M and APOLD1); 6) astrocytes (expressing GFAP and SOX9); and 7) oligodendrocytes (expressing CNP, MBP, and PLP1). Single-cell data analysis was performed as previously described by Yuan *et al^1^*. Single-cell RNA-seq analysis was performed using the "Seurat" R package (version 4.0.2).

**4. Lentiviral infection**

Lentiviruses carrying EGFRvIII (NM_001346941.2) and MDK (NM_010784.5) were transfected. The lentiviral plasmids carrying EGFRvIII and MDK were synthesized by Shanghai GenePharma Co., Ltd and Shanghai hanyinbt Co., Ltd, respectively.

The lentivirus transfection steps were as follows: lentivirus transfection was performed when the cell fusion degree reached 80-90%. 500μL serum-free medium was added to a 12-well plate, and an appropriate volume of EGFRvIII/MDK lentivirus or control lentivirus was added to each well. The plate was placed in a cell culture incubator and incubated for 12 hours. The medium was then replaced with complete medium, and the cells were further cultured for 48-72 hours before being passaged. Stable transfected cell lines were selected using puromycin and observed under a fluorescence microscope.

**5. Cell transfection with siRNA**

SiRNA targeting c-Fos was purchased from Shanghai GenePharma Co., Ltd. The siRNA powder was dissolved in ultrapure water ddH2O to prepare a 20μM solution. The siRNA transfection process is as follows: (1) Add 2ug of DNA to 200uL of jetPRIME® buffer. Gently vortex to mix. (2) Add 4uL of jetPRIME®, vortex for 10s, and let stand. (3) Incubate at room temperature for 10 min. (4) Add 200uL of the transfection complex to the cells containing serum. (5) Gently shake the culture dish to evenly distribute the transfection complex into the cells. (6) If necessary, replace with complete culture medium 4 hours after transfection and continue to culture. (7) Transfection was analyzed after 24 hours or according to experience.

**6. Western blotting**

After completing the gel casting, SDS-PAGE was performed until the sample ran to the green line at the bottom. Then, a wet transfer method (300mA, 90min) was used, followed by blocking with 5% BSA for 1 hour. The primary antibody, which was pre-prepared, was added and incubated overnight at 4°C. Antibody information is shown in the Supplementary Table S1. The membrane was washed three times with TBST, and then the corresponding secondary antibody was added and incubated at room temperature for 1 hour. The membrane was washed three times with TBST again. Enhanced chemiluminescence (ECL) was used for detection, and the G-box imaging system was used for exposure.

**7. qPCR**

After RNA extraction, cDNA was synthesized using a cDNA synthesis kit (TransGen Biotech, AE311), followed by Real-time PCR amplification. The qPCR primers were designed and synthesized by Sangon Biotech (Shanghai) Co.,Ltd., and the primer sequences are shown in the Supplementary Table S3. After PCR amplification, the real-time fluorescence quantitative PCR instrument automatically analyzed the results. The 2-△△CT method was used to analyze the expression differences of the target gene between the control group and each test group. The formula for calculation is as follows: △Ct=Ct target gene - Ct internal reference, then the average value of △Ct control group was obtained, which was denoted as △Ct control average. The △Ct of each group was subtracted from △Ct control average to obtain the △△Ct value, that is, △△Ct=△Ct sample - △Ct control average, and then the 2-△△CT value of each group was calculated, which represents the relative expression level of the gene in each group.

**8. Enzyme linked immunosorbent assay（ELISA）**

The Human Midkine ELISA Kit (EK1253) and Human CXCL1/GRO-α ELISA Kit (EK196) were purchased from Hangzhou Lianke Biotechnology Co., Ltd. After 48 hours of cell culture, the supernatant from each group was collected. Blank wells were set up, and 100μL of diluted supernatant samples and standards were added to the enzyme-linked immunosorbent assay (ELISA) plate, followed by incubation at 37°C for 90 minutes. The plate was washed three times, and 100μL of biotin-labeled human MDK/CXCL1 antibody was added to each well, followed by incubation at 37°C for 60 minutes. After another three washes, 100μL of streptavidin-peroxidase complex was added to each well (except the blank wells), and the plate was incubated at 37°C for 30 minutes. The plate was then washed four times, and 100μL of tetramethylbenzidine (TMB) substrate solution was added to each well, followed by a 15-minute reaction at 37°C. The reaction was stopped by adding stop solution, and the absorbance was immediately measured at a wavelength of 450 nm. The standard curve data were fitted using SPSS to generate a regression equation for concentration-absorbance, and the MDK/CXCL1 concentrations of the samples were calculated accordingly.

**9. Cell Proliferation Assay with Cell Counting Kit-8 (CCK-8)**

The CCK-8 (BS350A) was purchased from Biosharp, a brand of Lianjieke Technology Co., Ltd. First, cells were seeded into a 96-well plate and cultured until adherence. Next, different concentrations of drugs or treatment factors were added, and the cells were further cultured for a specified period. Then, CCK-8 reagent was added to each well, followed by incubation for 1.5 hours to allow viable cells to reduce the reagent to an orange-yellow formazan product. Finally, the absorbance (OD value) was measured at a wavelength of 450 nm using a microplate reader, and the cell proliferation rate was calculated to evaluate cell viability and drug effects.

**10. High-throughput sequencing**

The preparation of transcriptome and proteome libraries and sequencing process were performed by LC-Bio Technology CO., Ltd. (Hangzhou, China). Total RNA was extracted using TRIzol (Invitrogen, USA), and RNA deep sequencing was performed by illumina Novaseq™ 6000. Sequencing results were obtained as FPKM (fragments per kilobase exon per million reads) for each transcript. During the data analysis phase, gene expression profiles are obtained through quality control, sequence alignment, and gene expression quantification. Subsequently, differential expression analysis is performed to identify genes with significant changes. Finally, by integrating functional annotation and pathway enrichment analysis, the biological functions of the differentially expressed genes and the signaling pathways they are involved in are elucidated.

**11. Immunohistochemistry (IHC) and immunofluorescence (IF) staining**

IHC and IF staining were performed as previously described by Li *et al*^2^. IHC staining was assessed by the immunoreactive score (IRS) method^3^.

**12. Chromatin immunoprecipitation (ChIP)**

Predict the binding site sequence of the transcription factor (p-)c-Fos in the target gene MDK promoter region: (1) Use NCBI to obtain the potential promoter region base sequence of the target gene MDK; (2) Use the JASPAR database to query the transcription factor binding site (TFBS) information of c-Fos; (3) Use the JASPAR database to predict the binding site sequence of the transcription factor c-Fos in the target gene promoter region. Total 26 putative sites were predicted with relative profile score threshold 70% (Supplementary Table S3). Then, we performed primer design for the putative site with the highest relative scores (Supplementary Table S4).

The experimental steps are as follows: First, perform formaldehyde crosslinking and sonication of the cells. After sonication, centrifuge at 12000rpm, 4°C for 10 minutes. Remove the insoluble material and take the supernatant. Take 40μl of the sonication product as input, add 10μL of 5* reducing protein loading buffer, heat denatured and perform WB detection to confirm the presence of the target protein in the sample. Take 100μl and add 900μl of ChIP Dilution Buffer containing 1mM-PMSF and 20μl of 50×PIC (cocktail). Then add 60μl of Protein A+G Agarose/Salmon Sperm DNA to each tube. Mix well at 4°C for 1 hour. Divide the sample into two 1.5mL EP tubes, add 1μg of the target protein IP antibody to one tube, and add 1μg of corresponding species IgG to the other tube. Mix well at 4°C overnight. Then perform immunocomplex precipitation and washing, recover DNA samples using a centrifuge column. Regarding the obtained DNA, we initially utilized databases such as JASPAR to predict transcription factor binding sites. Based on these binding sites, primers were designed and synthesized. Subsequently, the accuracy of the fragment size was verified using real-time PCR and agarose gel electrophoresis.

**13. Co-immunoprecipitation (Co-IP)**

First, extract the cell protein, denature it and use it for input experiment, that is, WB detection of the target protein. Then, start the Co-IP experiment: (1) Add 1.0μg IgG (same species as the IP antibody source) and 20μL protein A/G beads to the negative control (IgG) group protein supernatant, and directly add 20μL protein A/G beads to the experimental group. Incubate at 4℃ with shaking for 1 hour. (2) After centrifugation, take the supernatant and add 1-10μL (0.2-2μg) antibody, then incubate overnight at 4℃. (3) Add 80μL protein A/G beads, mix well, and incubate at 4℃ for 2 hours. (4) Centrifuge and carefully remove the supernatant, being careful not to suck up the beads at the bottom, and collect the immune precipitation complex. (5) Wash the immune precipitation complex with 1ml pre-cooled IP lysis buffer, being careful to discard the supernatant after each wash. (6) After the final wash, remove the supernatant as much as possible, then add 80μL 1× reducing loading buffer, boil for 10 minutes in boiling water, centrifuge at 4℃ and 1000g for 5 minutes, and take the supernatant. Label as IP group, and use the prepared protein sample for WB detection of the target protein.

**14. Intracranial implantation model of glioma**

This experiment used 4-6 week old female C57BL/6 mice purchased from GemPharmatech Co.,Ltd, with animal qualification certificate number SCXK (Su) 2018-0008. Animal experiments strictly followed the principles of animal in vivo experiment safety and animal experimental ethics approved by Anhui Medical University (LLSC20240354). According to different treatments, they were divided into control group, MDK overexpression group, and MDK overexpression combined with iMDK (MDK inhibitor) group. The inhibitor iMDK was injected intraperitoneally (9mg/kg/day), and the drug was dissolved in DMSO and diluted with a solvent. The dilution scheme was: 10% DMSO + 40% PEG300 + 5% Tween-80 + 45% Saline.

The experimental steps are as follows: first, resuspend 3-5×105 GL261 cells infected with lentivirus expressing luciferase in 5μL serum-free DMEM solution and place on ice. Then, anesthetize the mice with isoflurane and place them on a stereotactic device for fixation. Disinfect the head skin with iodine, fully exposing the central area of the skull, positioning the anterior fontanelle, and drilling the hole 1 mm anterior to the anterior fontanelle and 2 mm to the right of the midline. After positioning, use a micro skull drill to drill the hole, use a micro syringe to extract 5μL of cell suspension, fix the micro syringe on an automatic micro syringe pump, slowly lower the three-dimensional positioning arm to the needle, move down 4 mm from the drilling hole, then move the needle back 1 mm and start injection. After the injection is complete, leave the needle in the skull for 1 minute. Slowly withdraw the needle. After the operation, suture the wound, punch the ear for numbering, and place it on a warming pad. After awakening, put the mouse back in the cage. After the inoculation is completed, use in vivo imaging technology to detect the growth of tumors in the mouse skull every 7 days.

**Supplementary Figure and Table Legends**

**Supplement Tables:**

**Table S1** Antibody information list.

**Table S2** Primer sequences.

**Table S3** Total 26 putative sites were predicted with relative profile score threshold 70%.

**Table S4** (p-)c-Fos promoter region binding site prediction and primer design.

**Table S5** Experimental protocol for flow cytometry.

**Supplement Figures:**

**Fig. S1 Distribution of different EGFR mutation types in patients with gliomas.** (A, B) The distribution of different EGFR mutations in brain glioma patients is shown.

**Fig. S2 Cell-cell communication analysis was performed on single-cell data from EGFRvIII(-) and EGFRvIII(+) GBM patients.**

(A, C) Outgoing and incoming communication patterns were analyzed in the single-cell data of EGFRvIII(-) (n=4) and EGFRvIII(+) (n=2) GBM patients. (B, D) Cell-cell interaction and social network analysis were conducted on the single-cell data of EGFRvIII(-) and EGFRvIII(+) GBM patients.

**Fig. S3 Analysis of the expression distribution, prognostic characteristics, and correlation between c-Fos and MDK in GBM using public databases.**

(A, E) Expression distribution of c-Fos and MDK in different WHO grades from TCGA, CGGA, and Renbrant databases. (B, F) Expression distribution of c-Fos and MDK in different molecular subtypes from TCGA and CGGA databases. (C, G) Expression distribution of c-Fos and MDK in different GBM subtypes from TCGA and CGGA databases. (D, H) Prognostic characteristics of c-Fos and MDK in primary glioma and GBM from TCGA and CGGA databases. (I) The correlation between c-Fos and MDK expression in glioma and GBM from TCGA and CGGA databases. *, **, *** and **** indicate P < 0.05, P < 0.01, P < 0.001 and P < 0.0001, respectively. Unpaired two-tailed Student’s t test. The overall survival of gliomas was analyzed for prognosis using the Kaplan-Meier method.

**Fig. S4 Analysis of the expression characteristics and correlation between c-Fos and MDK in GBM patients using tissue microarrays.**

(A) Immunohistochemical staining was performed to analyze the expression characteristics of c-Fos and MDK in different WHO grade gliomas. (B-E) Tissue chips were used for immunohistochemical staining (including c-Fos and MDK), and the results were analyzed(nontumor(n=5), WHO grade 2(n=30), WHO grade 3(n=15), WHO grade 4(n=40)). All data were shown as mean ± SD (bar plots). *, **, *** and **** indicate P < 0.05, P < 0.01, P < 0.001 and P < 0.0001, respectively. Unpaired two-tailed Student’s t test.

**Fig. S5 Inter-cellular communication analysis shows the receptor-ligand relationships in the MDK signaling pathway in EGFRvIII-mutant GBM.**

(A) tSNE plot of EGFRvIII(+) GBMs (n=2). (B) Communication network analysis of the MDK signaling pathway between different cell subpopulations. (C) Calculation and visualization of the contribution of each ligand-receptor pair to the entire signaling pathway. (D) Identification of signal transduction roles in the intercellular communication network, including primary senders, receivers, mediators, and influencers. (E, F) Expression levels and tSNE plot distribution of the MDK ligand and its receptors NCL/PTPRZ1/LRP1 in different cell subpopulations of EGFRvIII(+) glioblastoma. *, **, *** and **** indicate P < 0.05, P < 0.01, P < 0.001 and P < 0.0001, respectively. Unpaired two-tailed Student’s t test.

**Fig. S6 MDK secreted by GBM cells promotes macrophage secretion of the immunosuppressive cytokine CXCL1.**

(A) Top 100 DEGs between siCon (n=3) and siMDK groups (n=3). (B) Top 10 DEGs between siCon and siMDK groups. (C, D) GO and KEGG pathway analysis of differentially expressed genes between siCon and siMDK groups. (E) Immunofluorescence staining detecting the expression and distribution characteristics of MDK, CD206, and CXCL1 in patients with EGFRvIII (-) (n=3) and EGFRvIII (+) (n=3) GBM.

**Fig. S7 CXCL1 secreted by macrophages promotes the proliferation and migration of GBM cells.**

(A) CXCL1 mRNA and protein expression levels were evaluated by qPCR (n=3) and Elisa (n=3) in human THP1 cell line with siCon and siCXCL1 group, respectively. (B) The CCK-8 assay (n=3) was used to evaluate the cell proliferation capacity of U87MG, U251MG, and T98MG under different concentrations of CXCL1 protein (0 pg/ml, 250 pg/ml, and 500 pg/ml). (C-E) The chemotactic migration capabilities of U87MG, U251MG, and T98MG cells were evaluated under different concentrations of CXCL1 protein (0 pg/ml, 250 pg/ml, and 500 pg/ml), as well as under co-culture conditions with siCon and CXCL1 THP1 treatments (n=3). All data were shown as mean ± SD (bar plots). *, **, *** and **** indicate P < 0.05, P < 0.01, P < 0.001 and P < 0.0001, respectively. Unpaired two-tailed Student’s t test.

**Fig. S8 In vivo experiments to validate the effect of targeted MDK signaling pathway on tumor growth.**

(A) Bioluminescence images of GBM-bearing mice in control (n=10), MDK overexpression (n=10), and MDK overexpression plus iMDK groups (n=10) groups (at 7, 14, and 21days). (B) Quantitative analysis of tumor burden based on bioluminescence intensity. Two-way analysis of variance (two-way ANOVA).

**Fig. S9 Targeting the MDK signaling pathway in GBM can affect tumor growth and the immune microenvironment.**

HE staining of intracranial tumors in control (n=3), MDK overexpression (n=3), and MDK overexpression plus iMDK groups (n=3). Immunohistochemistry analysis of the expression characteristics of MDK, CXCL1, Ki67, CD3, CD4, CD8, CD68, iNOS, CD206, PD1, PD-L1, and Foxp3 in control, MDK overexpression, and MDK overexpression plus iMDK groups.

**Fig. S10 Targeting the MDK signaling pathway in GBM can affect the immune microenvironment.**

Flow cytometry analysis of the distribution characteristics of immune cell subpopulations (M1 and M2 type macrophages, Tregs, CD4+ and CD8+ T cells) in control (n=3), MDK overexpression (n=3), and MDK overexpression plus iMDK groups (n=3).

**Reference**

1. Yuan F, Cai X, Cong Z, Wang Y, Geng Y, Aili Y*, et al.* Roles of the m(6)A Modification of RNA in the Glioblastoma Microenvironment as Revealed by Single-Cell Analyses. *Front Immunol* 2022, **13:** 798583.

2. Li T, Mehraein-Ghomi F, Forbes ME, Namjoshi SV, Ballard EA, Song Q*, et al.* HSP90-CDC37 functions as a chaperone for the oncogenic FGFR3-TACC3 fusion. *Mol Ther* 2022, **30**(4)**:** 1610-1627.

3. Ilie MD, Tabarin A, Vasiljevic A, Bonneville JF, Moreau-Grangé L, Schillo F*, et al.* Predictive Factors of Somatostatin Receptor Ligand Response in Acromegaly-A Prospective Study. *J Clin Endocrinol Metab* 2022, **107**(11)**:** 2982-2991.
