# Supplementary material for: EGFRvIII-positive glioblastoma contributes to immune escape and malignant progression via the c-Fos-MDK-LRP1 axis
Source: Cell Death Dis. 2025 Jun 17;16(1):453. doi: 10.1038/s41419-025-07771-1 (PMC12174314; doi:10.1038/s41419-025-07771-1)
Supplement: Supplementary file 2 — Supplementary Table S1 [file 41419_2025_7771_MOESM2_ESM.docx]

**Supplementary Table S1 Antibody information list**

| **Antibody** | **Production company** | **Product number** |
| --- | --- | --- |
| EGF Receptor vIII (D6T2Q) XP® Rabbit mAb | CST | 64952T |
| Anti-Midkine [EP1143Y] | Abcam | ab52637 |
| CD206 Mouse Polyclonal Antibody | Proteintech | 60143-1-Ig |
| Midkine (MDK) Mouse Monoclonal Antibody [Clone ID: OTI8C6] | OriGene | TA807775 |
| Anti-LRP1 [EPR3724] | Abcam | ab92544 |
| c-Fos (9F6) Rabbit mAb | CST | 2250T |
| c-Fos (E7L5L) Mouse mAb | CST | 74620S |
| Anti-c-Fos (phospho T325) | Abcam | ab27793 |
| Anti- c-Fos Rabbit pAb | Servicebio | GB114125 |
| c-Fos (6-2H-2F) | Santa | sc-447 |
| p44/42 MAPK (Erk1/2) Antibody | CST | 9102S |
| Phospho-p44/42 MAPK (Erk1/2) (Thr202/Tyr204) Antibody | CST | 9101S |
| Anti -Phospho-ERK1 (T202/Y204) + ERK2 (T185/Y187) Rabbit pAb | Servicebio | GB11004 |
| human mldkine | Absin | abs00930 |
| CXCL1 Polyclonal antibody | PTG | 12335-1-AP |
| Anti -beta Actin Rabbit pAb | Servicebio | GB11001 |
| CD3ε (E4T1B) XP® Rabbit mAb | CST | 78588T |
| CD4 (D7D2Z) Rabbit mAb | CST | 25229T |
| CD8α (D4W2Z) XP Rabbit mAb (Mouse Specific) | CST | 98941T |
| PA-ANTI-MO/RT FOXP3 FJK-16S PUR | Invitrogen | 14-5773-80 |
| CD68 (E3O7V) Rabbit mAb | CST | 28058-1-AP |
| INOS Polyclonal Antibody | Proteintech | 22226-1-AP |
| CD163 Rabbit Polyclonal Antibody | Proteintech | 16646-1-AP |
| PD-L1/CD274 Mouse Monoclonal Antibody | Proteintech | 66248-1-Ig |
| Ki-67 Polyclonal Antibody | ImmunoWay | YT2467 |
| ANTI-MO CD3 17A2 FITC | eBioscience | 11-0032-80 |
| ANTI-MO CD4 RM4-5 PERCP-CYN5.5 | eBioscience | 45-0042-80 |
| ANTI-MO CD8A 53-6.7 APC | eBioscience | 17-0081-81 |
| ANTI-M/R FOXP3 FJK-16S PE | eBioscience | 12-5773-82 |
| FIXABLE VIABILITY DYE EF450 | eBioscience | 65-0863-14 |
| RAT IGG2BK ISOCTRL FITC | eBioscience | 11-4031-81 |
| RAT IGG2AK ISO CTRL EBR2A PE | eBioscience | 12-4321-80 |
| RT IGG2AK ISO CTL PERCP-CYN5.5 | eBioscience | 45-4321-80 |
| RAT IGG2AK ISO CTRL EBR2A APC | eBioscience | 17-4321-81 |
| ANTI-MO CD163 TNKUPJ APC | eBioscience | 17-1631-80 |
| ANTI-MO CD86 GL1 PE | eBioscience | 12-0862-81 |
| ANTI-MO CD11B M1/70 FITC | eBioscience | 11-0112-81 |
| FOXP3/TRN FACTOR STAIN BUFFER | eBioscience | 00-5523-00 |
| APC/Fire™ 750 anti-mouse CD45 | BioLegend | 103153 |
| ANTI-M F4/80 AG BM8 PCP-CYN5.5 | eBioscience | 45-4801-80 |
| ULTRACOMP PLUS BEADS, 100 TESTS | eBioscience | 01-3333-42 |
| Ms CD45 BV510 30-F11 50ug | BD Pharmingen | 563891 |
