# Supplementary material for: EGFRvIII-positive glioblastoma contributes to immune escape and malignant progression via the c-Fos-MDK-LRP1 axis
Source: Cell Death Dis. 2025 Jun 17;16(1):453. doi: 10.1038/s41419-025-07771-1 (PMC12174314; doi:10.1038/s41419-025-07771-1)
Supplement: Supplementary file 3 — Supplementary Table S2 [file 41419_2025_7771_MOESM3_ESM.docx]

**Supplementary Table S2 The primer sequences for qPCR**

| **Primer** | **Sequence（5'-3'）** |
| --- | --- |
| human-EGFRvIII-F | GTGGATGCCGACGAGTACCTC |
| human-EGFRvIII-R | ACAGCTTTGCAGCCCATTTCTAT |
| Human-MDK-F | AAGGATTGCGGCGTGGGTTTC |
| Human-MDK-R | TGGCGGACTTTGGTGCCTGTG |
| human-c-Fos-F | CTCCAGTGCCAACTTCATTCCC |
| human-c-Fos-R | GCCATCTTATTCCTTTCCCTTCG |
| mouse -FOS-F | ACCCTTTGATGACTTCTTGTTTCC |
| mouse -FOS-R | TTGCTGTGCAGAGGCTCCC |
| mouse -MDK-F | GCCGACTGCAAATACAAGTTTGAGA |
| mouse-MDK-R | GGGCTTAGTCACGCGGATGGT |
| human-ERK1-F | AAAGCCAGCAGCTGAGCAAT |
| human-ERK1-R  human-CXCL1-F  human-CXCL1-R | TTAAGGTCGCAGGTGGTGTT  AGCTTGCCTCAATCCTGCATCC  TCCTTCAGGAACAGCCACCAGT |
| Human-GAPDH-F | AATCCCATCACCATCTTCCA |
| Human-GAPDH-R | AAATGAGCCCCAGCCTTCT |
