# Supplementary material for: EGFRvIII-positive glioblastoma contributes to immune escape and malignant progression via the c-Fos-MDK-LRP1 axis
Source: Cell Death Dis. 2025 Jun 17;16(1):453. doi: 10.1038/s41419-025-07771-1 (PMC12174314; doi:10.1038/s41419-025-07771-1)
Supplement: Supplementary file 4 — Supplementary Table S3 [file 41419_2025_7771_MOESM4_ESM.docx]

**Supplementary Table S3 Total 26 putative sites were predicted with relative profile score threshold 70%.**

| **Matrix ID** | **Name** | **Score** | **Relative score** | **Sequence ID** | **Start** | **End** | **Strand** | **Predicted sequence** |
| --- | --- | --- | --- | --- | --- | --- | --- | --- |
| **MA0476.1** | **MA0476.1.FOS** | **12.038263** | **0.9494942100956221** | **NC_000011.10:46378784-46380784** | **1740** | **1750** | **+** | **TGTGACTCAGT** |
| **MA0476.1** | **MA0476.1.FOS** | **11.816569** | **0.9465374497384785** | **NC_000011.10:46378784-46380784** | **1740** | **1750** | **-** | **ACTGAGTCACA** |
| **MA0476.1** | **MA0476.1.FOS** | **2.5403805** | **0.8228197651284557** | **NC_000011.10:46378784-46380784** | **78** | **88** | **-** | **TCTTACTCCTG** |
| **MA0476.1** | **MA0476.1.FOS** | **2.1824481** | **0.8180459771291919** | **NC_000011.10:46378784-46380784** | **1128** | **1138** | **-** | **GCTGGATCATC** |
| **MA0476.1** | **MA0476.1.FOS** | **2.0973501** | **0.81691101414154** | **NC_000011.10:46378784-46380784** | **904** | **914** | **-** | **GCTGGCTCAGG** |
| **MA0476.1** | **MA0476.1.FOS** | **-0.44713664** | **0.7829748776872213** | **NC_000011.10:46378784-46380784** | **1128** | **1138** | **+** | **GATGATCCAGC** |
| **MA0476.1** | **MA0476.1.FOS** | **-1.5739132** | **0.767946918327091** | **NC_000011.10:46378784-46380784** | **636** | **646** | **+** | **GGTGGATCAGG** |
| **MA0476.1** | **MA0476.1.FOS** | **-1.8005086** | **0.7649247872664139** | **NC_000011.10:46378784-46380784** | **636** | **646** | **-** | **CCTGATCCACC** |
| **MA0476.1** | **MA0476.1.FOS** | **-1.8584669** | **0.7641517909394289** | **NC_000011.10:46378784-46380784** | **904** | **914** | **+** | **CCTGAGCCAGC** |
| **MA0476.1** | **MA0476.1.FOS** | **-3.362518** | **0.7440920728751548** | **NC_000011.10:46378784-46380784** | **87** | **6L** | **-** | **GCTCACACATC** |
| **MA0476.1** | **MA0476.1.FOS** | **-3.6078966** | **0.7408194293732973** | **NC_000011.10:46378784-46380784** | **1231** | **1241** | **+** | **ACTGCCACATT** |
| **MA0476.1** | **MA0476.1.FOS** | **-3.9825418** | **0.7358227392946968** | **NC_000011.10:46378784-46380784** | **554** | **564** | **-** | **GATGACAGATG** |
| **MA0476.1** | **MA0476.1.FOS** | **-4.1038246** | **0.7342051752916104** | **NC_000011.10:46378784-46380784** | **878** | **888** | **-** | **ACTGTGTCTTC** |
| **MA0476.1** | **MA0476.1.FOS** | **-4.3123183** | **0.7314244687515621** | **NC_000011.10:46378784-46380784** | **1323** | **1333** | **-** | **CCTGAGTCCAG** |
| **MA0476.1** | **MA0476.1.FOS** | **-5.024235** | **0.7219295505432145** | **NC_000011.10:46378784-46380784** | **1323** | **1333** | **+** | **CTGGACTCAGG** |
| **MA0476.1** | **MA0476.1.FOS** | **-5.2457933** | **0.7189745963228357** | **NC_000011.10:46378784-46380784** | **58** | **68** | **-** | **TGTGGGTCTCT** |
| **MA0476.1** | **MA0476.1.FOS** | **-5.2836537** | **0.7184696475447573** | **NC_000011.10:46378784-46380784** | **1341** | **1351** | **+** | **GGGGCCTCACC** |
| **MA0476.1** | **MA0476.1.FOS** | **-5.4135704** | **0.7167369327834715** | **NC_000011.10:46378784-46380784** | **1379** | **1389** | **-** | **TGTGAGCCTCC** |
| **MA0476.1** | **MA0476.1.FOS** | **-5.473889** | **0.7159324578457448** | **NC_000011.10:46378784-46380784** | **1707** | **1717** | **-** | **TGAGAGTCCCG** |
| **MA0476.1** | **MA0476.1.FOS** | **-5.502895** | **0.7155456011577448** | **NC_000011.10:46378784-46380784** | **518** | **528** | **+** | **ATTTTGTCAGG** |
| **MA0476.1** | **MA0476.1.FOS** | **-5.611805** | **0.7140930538226163** | **NC_000011.10:46378784-46380784** | **1341** | **1351** | **-** | **GGTGAGGCCCC** |
| **MA0476.1** | **MA0476.1.FOS** | **-5.70262** | **0.7128818419732058** | **NC_000011.10:46378784-46380784** | **1593** | **1603** | **-** | **GCTGAGTTTCC** |
| **MA0476.1** | **MA0476.1.FOS** | **-6.20815** | **0.7061395270712344** | **NC_000011.10:46378784-46380784** | **623** | **633** | **-** | **TCTGTCTCCCA** |
| **MA0476.1** | **MA0476.1.FOS** | **-6.31557** | **0.7047068536001533** | **NC_000011.10:46378784-46380784** | **1379** | **1389** | **+** | **GGAGGCTCACA** |
| **MA0476.1** | **MA0476.1.FOS** | **-6.4063845** | **0.7034956481103792** | **NC_000011.10:46378784-46380784** | **132** | **142** | **-** | **GCTGGCCCACA** |
| **MA0476.1** | **MA0476.1.FOS** | **-6.612001** | **0.7007533156169427** | **NC_000011.10:46378784-46380784** | **58** | **68** | **+** | **AGAGACCCACA** |
