# Supplementary material for: EGFRvIII-positive glioblastoma contributes to immune escape and malignant progression via the c-Fos-MDK-LRP1 axis
Source: Cell Death Dis. 2025 Jun 17;16(1):453. doi: 10.1038/s41419-025-07771-1 (PMC12174314; doi:10.1038/s41419-025-07771-1)
Supplement: Supplementary file 5 — Supplementary Table S4 [file 41419_2025_7771_MOESM5_ESM.docx]

**Supplementary Table S4** **Transcription factor binding sites and Primer design**

cFOS→MDK，Human

>NC_000011.10:46378784-46380784 Homo sapiens chromosome 11, GRCh38.p14 Primary Assembly

GCTGCCAGAGAGCCCACAGGCTGTGGGGTGAGAGGCCCCTCCTCCGGTGTGCTCCAGAGAGACCCACAGAGGCAACTCAGGAGTAAGATGTGTGAGCGCACTCGTTTCAGGCCTGTGCTGGTGTACCCGGCTGTGGGCCAGCGTGTGAGCTCAGGGAAGGAGGGGTGGCCCCAGGAGGTCCAGCCCTGCCATGCCTCCTGCCCTCAGCTCCAGGAGCTGCACCGAGCTGGGGGCGACCTCATGCACCGAGACGAGCAGAGTCGCACGCTCCTGCACCACGCAGTCAGCACTGGCAGCAAGGATGTGGTCCGCTACCTGCTGGACCACGGTGAGCCGGGCAGTGGAGCCCAGGGCCTGGGGCCAAGGTGGGAGGAGGAGGGGCTGCCAGGCCTCTCTGACCACCACCTCCCCTTCTCCAGCCCCCCCAGAGATCCTTGATGCGGTGGAGGAAAAGTAAGTATCTGGGCAGTGCAGAACCGTGGTCACCCCGGAAACCACCCTTTTCCCCACCCCTCCCATTTTGTCAGGTCAGAGCCCATAAACTTCCTGGTCACATCTGTCATCCCCTGGGCCACCCCTATTGCCCCAGAGCCCTGAACTTCCTGCCCTTTCTGATGGCCCTTGGGAGACAGATGGGTGGATCAGGGGACGGGATGGGGTACACAGCCAGCCCCTGCTCCCCCAGCGGGGAGACCTGTTTGCACCAAGCAGCGGCCCTGGGCCAGCGCACCATCTGCCACTACATCGTGGAGGCCGGGGCCTCGCTCATGAAGACAGACCAGCAGGTGAGCAGACGGCAGGCAGGGAGCCCACGAGGGCACCAACCAAACCTTTCCCAAGGTCCTAGGCGGGAGCTGGGGCTGGGGGCTGTCCCTGGGAAGACACAGTCCAGACCCTGGGAAACCTGAGCCAGCAGGGGAGGAGCTGGTGGGCAGAGAGGCCTCCCTCCCTGACCAGGCCACAGGGAGGTAGAGCCCCTGCCTCTCAGCCTGCTAGGGGTTAGGCCTGCCTCTGGCCCCTGCTGATCGCAGCTCCGCCCTCCTCCAGGGCGACACTCCCCGGCAGCGGGCTGAGAAGGCTCAGGACACCGAGCTGGCCGCCTACCTGGAGAACCGGCAGCACTACCAGATGATCCAGCGGGAGGACCAGGAGACGGCTGTGTAGCGGGCCGCCCACGGGCAGCAGGAGGGACAATGCGGCCAGGGGACGAGCGCCTTCCTTGCCCACCTCACTGCCACATTCCAGTGGGACGGCCACGGGGGGACCTAGGCCCCAGGGAAAGAGCCCCATGCCGCCCCCTAAGGAGCCGCCCAGACCTAGGGCTGGACTCAGGAGCTGGGGGGGCCTCACCTGTTCCCCTGAGGACCCCGCCGGACCCGGAGGCTCACAGGGAACAAGACACGGCTGGGTTGGATATGCCTTTGCCGGGGTTCTGGGGCAGGGCGCTCCCTGGCCGCAGCAGATGCCCTCCCAGGAGTGGAGGGGCTGGAGAGGGGGAGGCCTTCGGGAAGAGGCTTCCTGGGCCCCCTGGTCTTCGGCCGGGTCCCCAGCCCCCGCTCCTGCCCCACCCCACCTCCTCCGGGCTTCCTCCCGGAAACTCAGCGCCTGCTGCACTTGCCTGCCCTGCCTTGCTTGGCACCCGCTCCGGCGACCCTCCCCGCTCCCCTGTCATTTCATCGCGGACTGTGCGGCCTGGGGGTGGGGGGCGGGACTCTCACGGTGACATGTTTACAGCTGGGTGTGACTCAGTAAAGTGGATTTTTTTTTCTTTTCTGCTTTTCTTCTTTTGCGGGGGAGGTCTAACAAGCAGCGGGGGCTGCGGGGTTGTCCTCGGGGTGGGGGACTGGACGCTGTCGACAGCACCTTCCTGGGGCCCCGGCTCCCGTTTGGTGGTTGGTCCCAGGGCCTGCCCGGTTCCTGACCTCTGCCCGGCGGCCGCGCTCGTCGGGGCCGGGGGCGGGGCCGATCCCTCCGGCTTCCCGCTTCCCGCGGAGAACAACA

| **Name** | | **Primer sequence (5'to3')** | **Fragment size (bp)** |
| --- | --- | --- | --- |
| Chip-qPCR | F | GCTCCCCTGTCATTTCATCG | 133 |
|  | R | CCGCAAAAGAAGAAAAGCAGAA |  |
