# Supplementary material for: EGFRvIII-positive glioblastoma contributes to immune escape and malignant progression via the c-Fos-MDK-LRP1 axis
Source: Cell Death Dis. 2025 Jun 17;16(1):453. doi: 10.1038/s41419-025-07771-1 (PMC12174314; doi:10.1038/s41419-025-07771-1)
Supplement: Supplementary file 6 — Supplementary Table S5 [file 41419_2025_7771_MOESM6_ESM.docx]

**Supplementary Table S4** **Experimental protocol for flow cytometry**

| **Subtype** | **Indicator** | **Fluorescein** | **Product number** |
| --- | --- | --- | --- |
| Panel 1 | CD45 | BV510 | 563891 |
|  | CD11B | FITC | 11-0112-81 |
|  | CD86 | PE | 12-0862-81 |
|  | CD163 | APC | 17-1631-80 |
|  | Live/Dead | APC-CY7 | 565388 |
| Panel 2 | CD45 | BV510 | 563891 |
|  | CD3 | FITC | 11-0032-80 |
|  | CD4 | PERCP-CY5.5 | 45-0042-80 |
|  | CD8 | APC | 17-0081-81 |
|  | FOXP3 | PE | 12-5773-82 |
|  | Live/Dead | APC-CY7 | 565388 |
