# Supplementary figures and images for: EGFRvIII-positive glioblastoma contributes to immune escape and malignant progression via the c-Fos-MDK-LRP1 axis
Source: Cell Death Dis. 2025 Jun 17;16(1):453. doi: 10.1038/s41419-025-07771-1 (PMC12174314; doi:10.1038/s41419-025-07771-1)

**Fig4A**




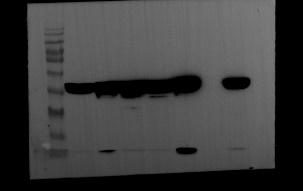

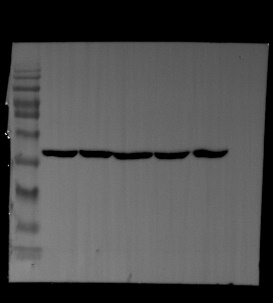


**Fig4C-U87**


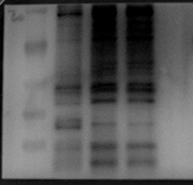

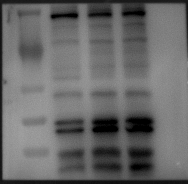

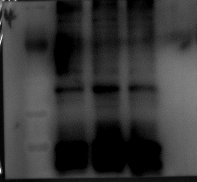

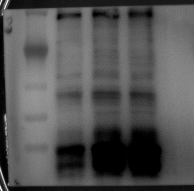

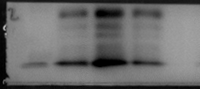


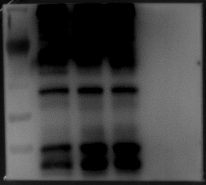


**Fig4C-U251**


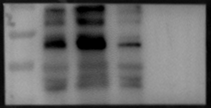

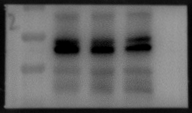

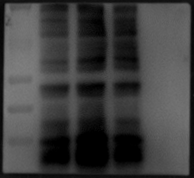

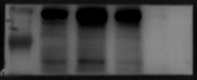

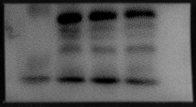


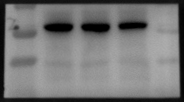


**Fig4C-T98**


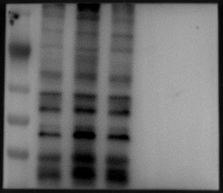

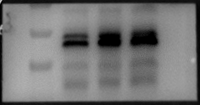

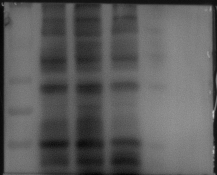

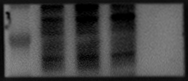

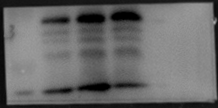


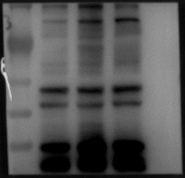


**Fig4G**

**
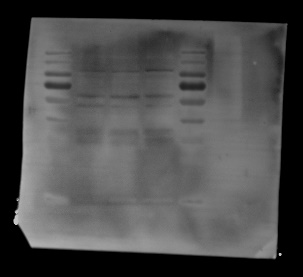
**

**Fig4H**

**
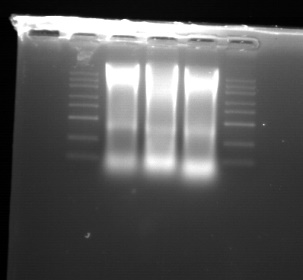
**

**Fig4I**

**
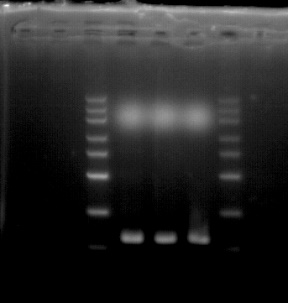
**

**Fig4J**

**

**

**Fig5A**

**
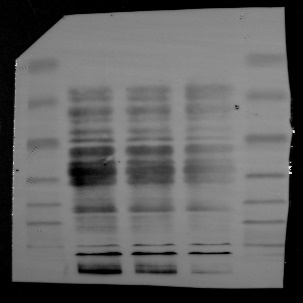
** **
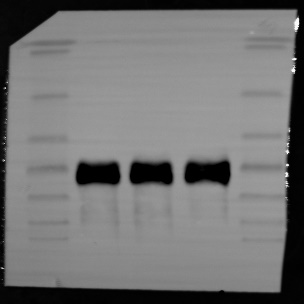
**
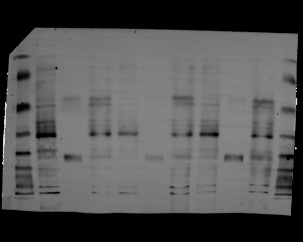

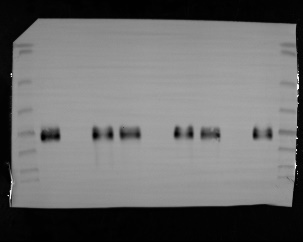


**Fig5B**


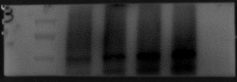

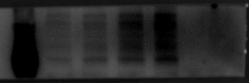

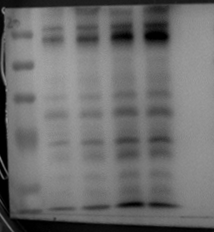

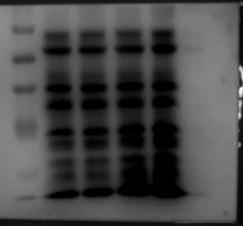


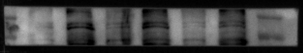

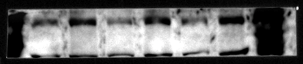

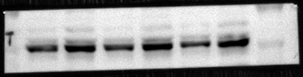

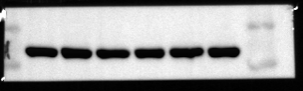

Supplement: Supplementary file 7 — Original Western Blots [file 41419_2025_7771_MOESM7_ESM.docx]

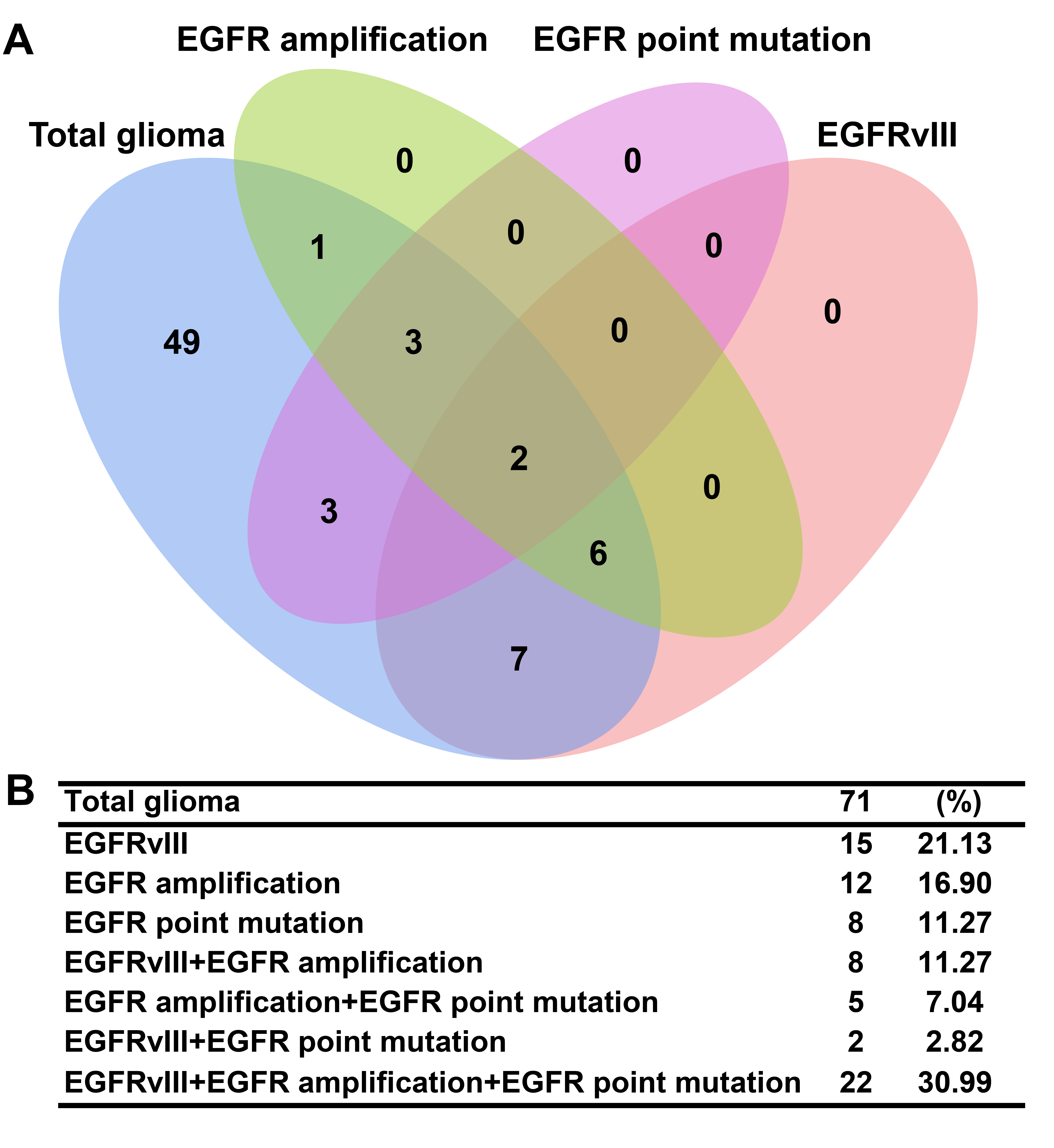

Supplement: Supplementary file 8 — sFigure 1 [file 41419_2025_7771_MOESM8_ESM.tif]

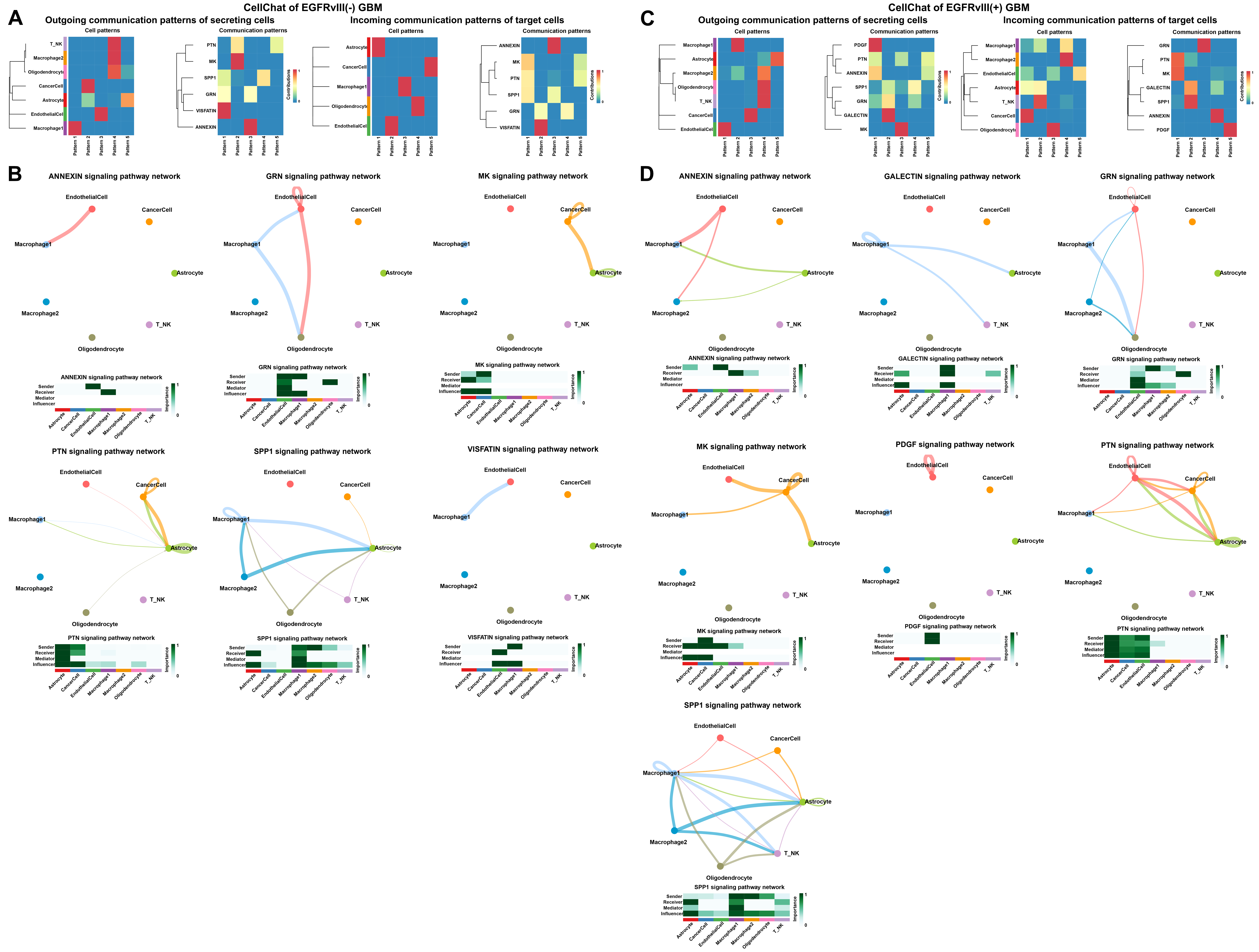

Supplement: Supplementary file 9 — sFigure 2 [file 41419_2025_7771_MOESM9_ESM.tif]

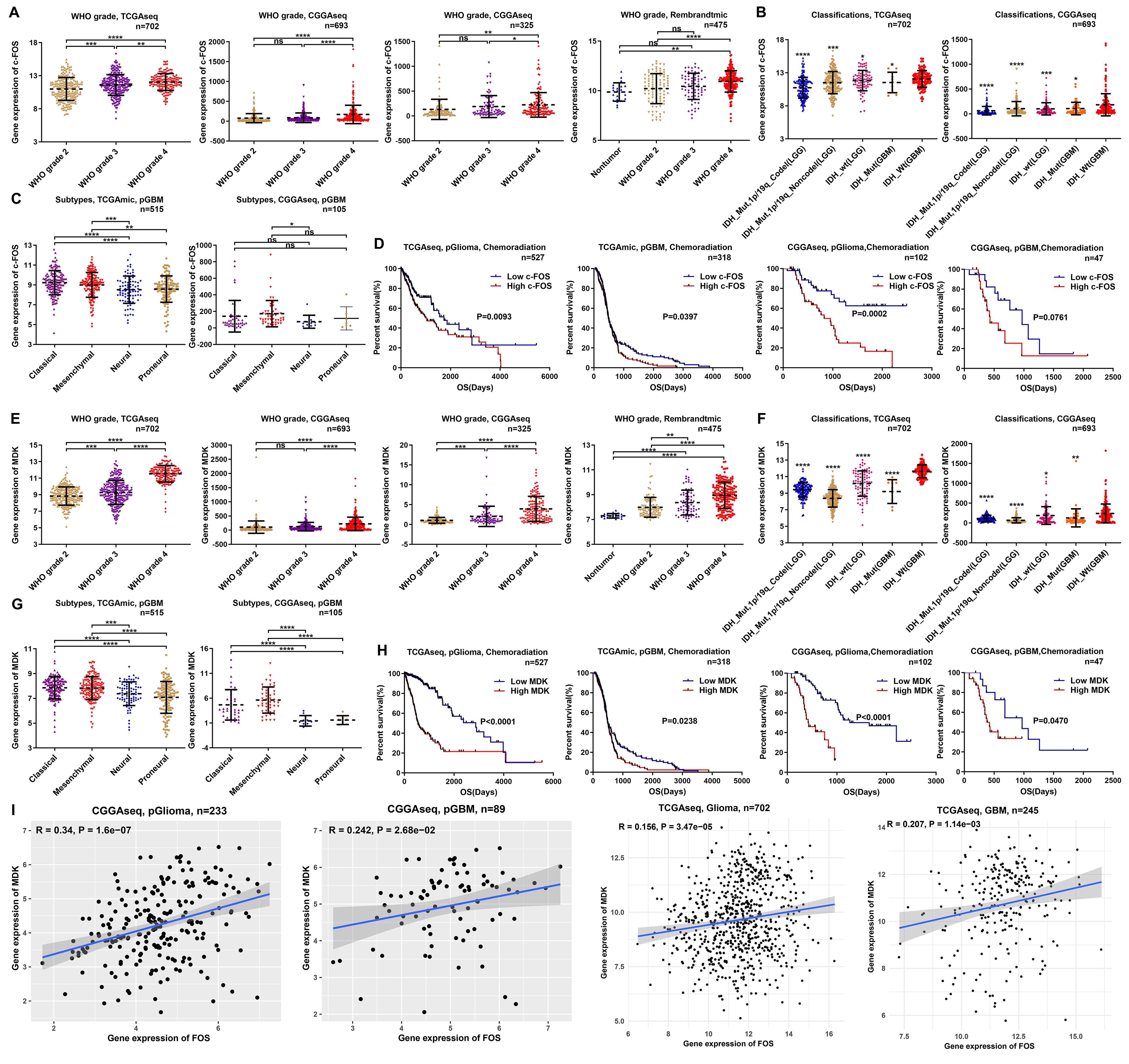

Supplement: Supplementary file 10 — sFigure 3 [file 41419_2025_7771_MOESM10_ESM.tif]

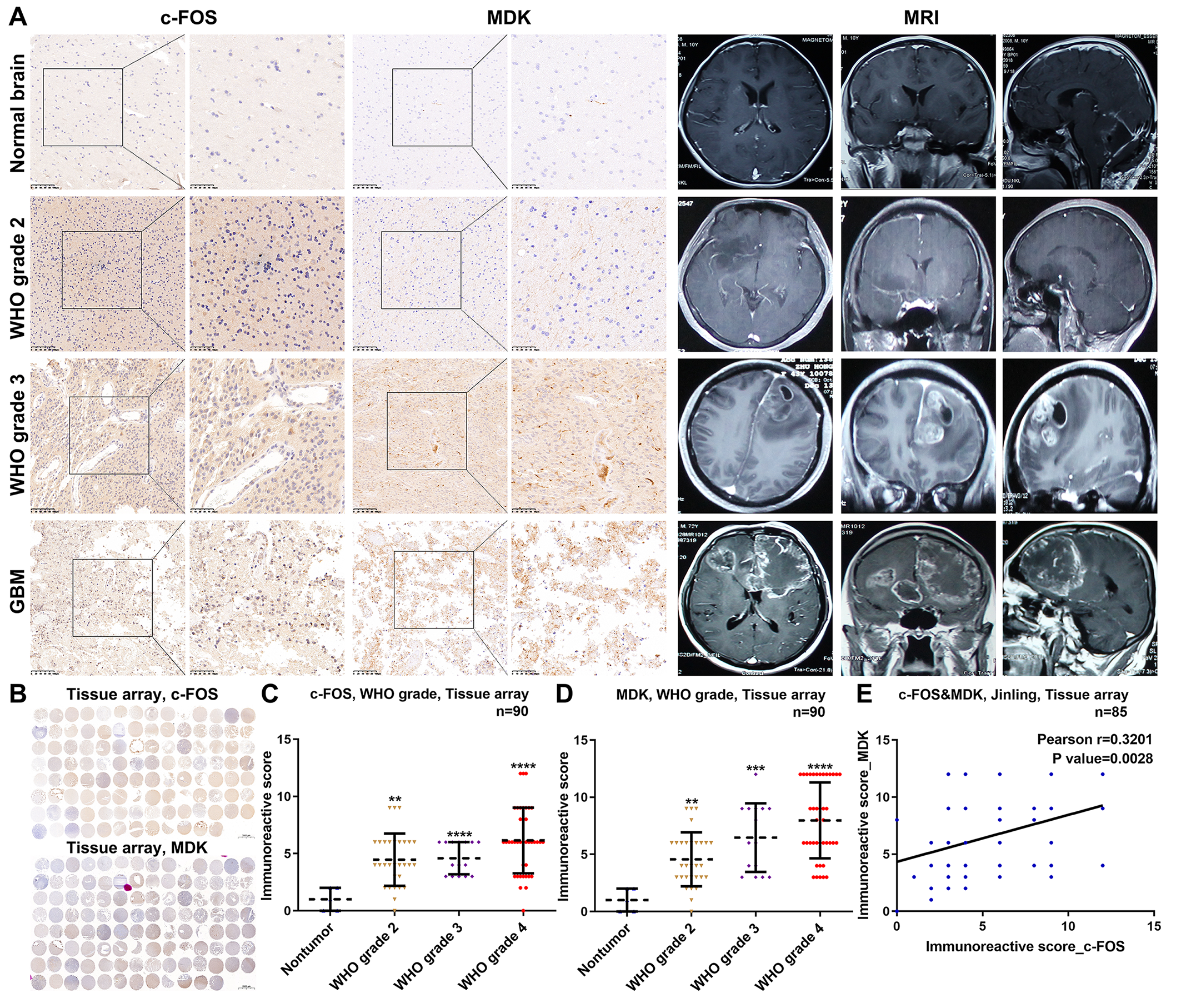

Supplement: Supplementary file 11 — sFigure 4 [file 41419_2025_7771_MOESM11_ESM.tif]

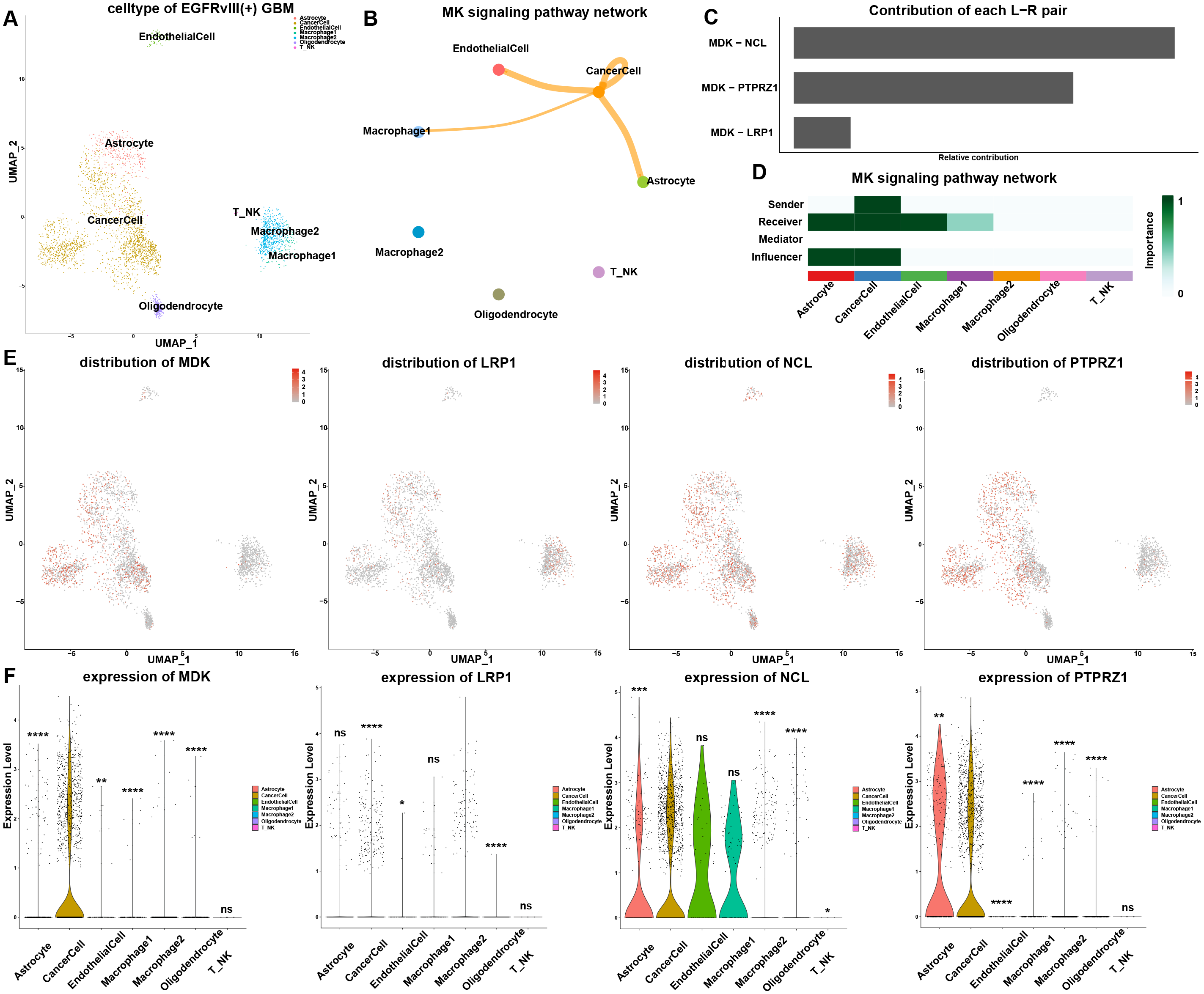

Supplement: Supplementary file 12 — sFigure 5 [file 41419_2025_7771_MOESM12_ESM.tif]

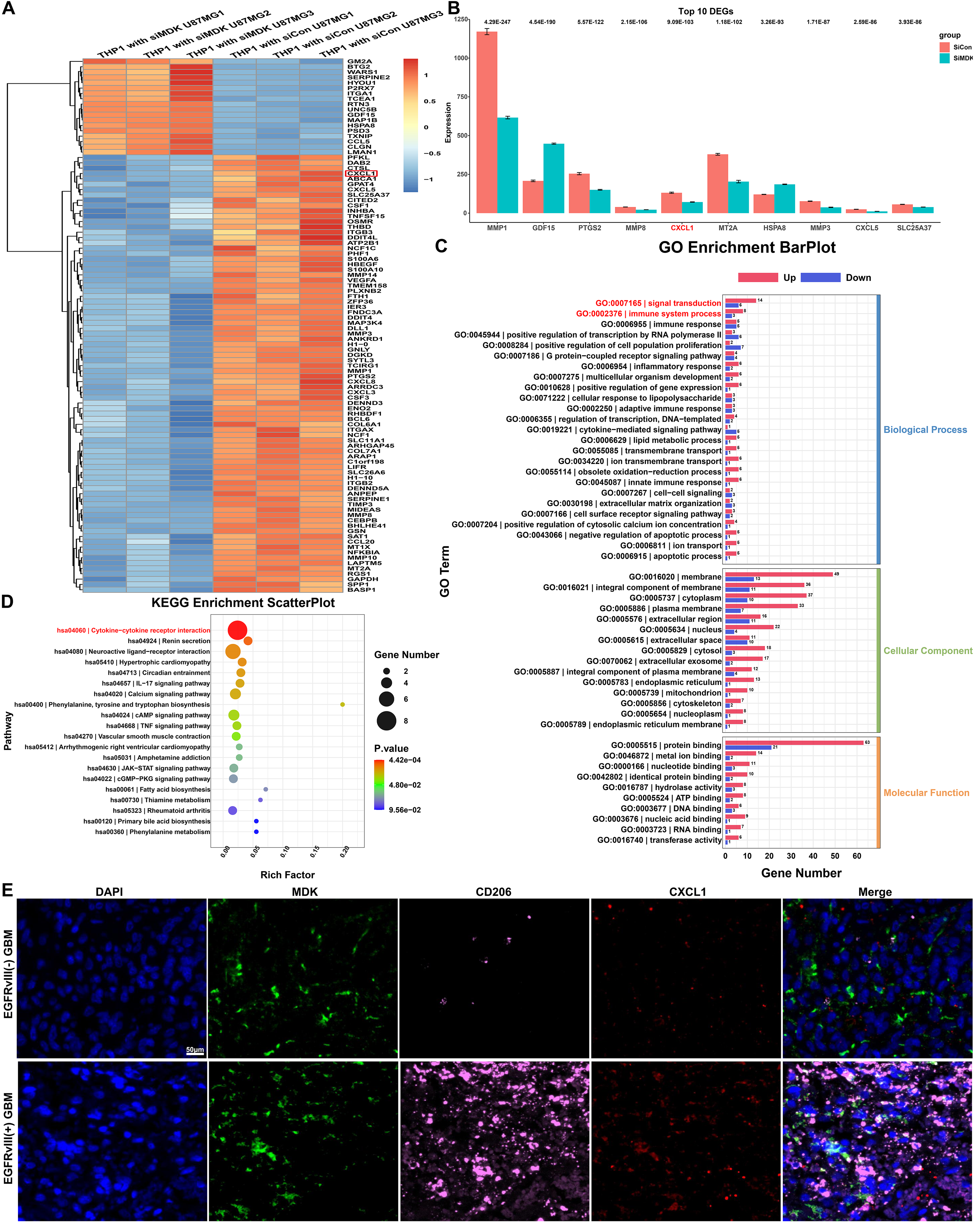

Supplement: Supplementary file 13 — sFigure 6 [file 41419_2025_7771_MOESM13_ESM.tif]

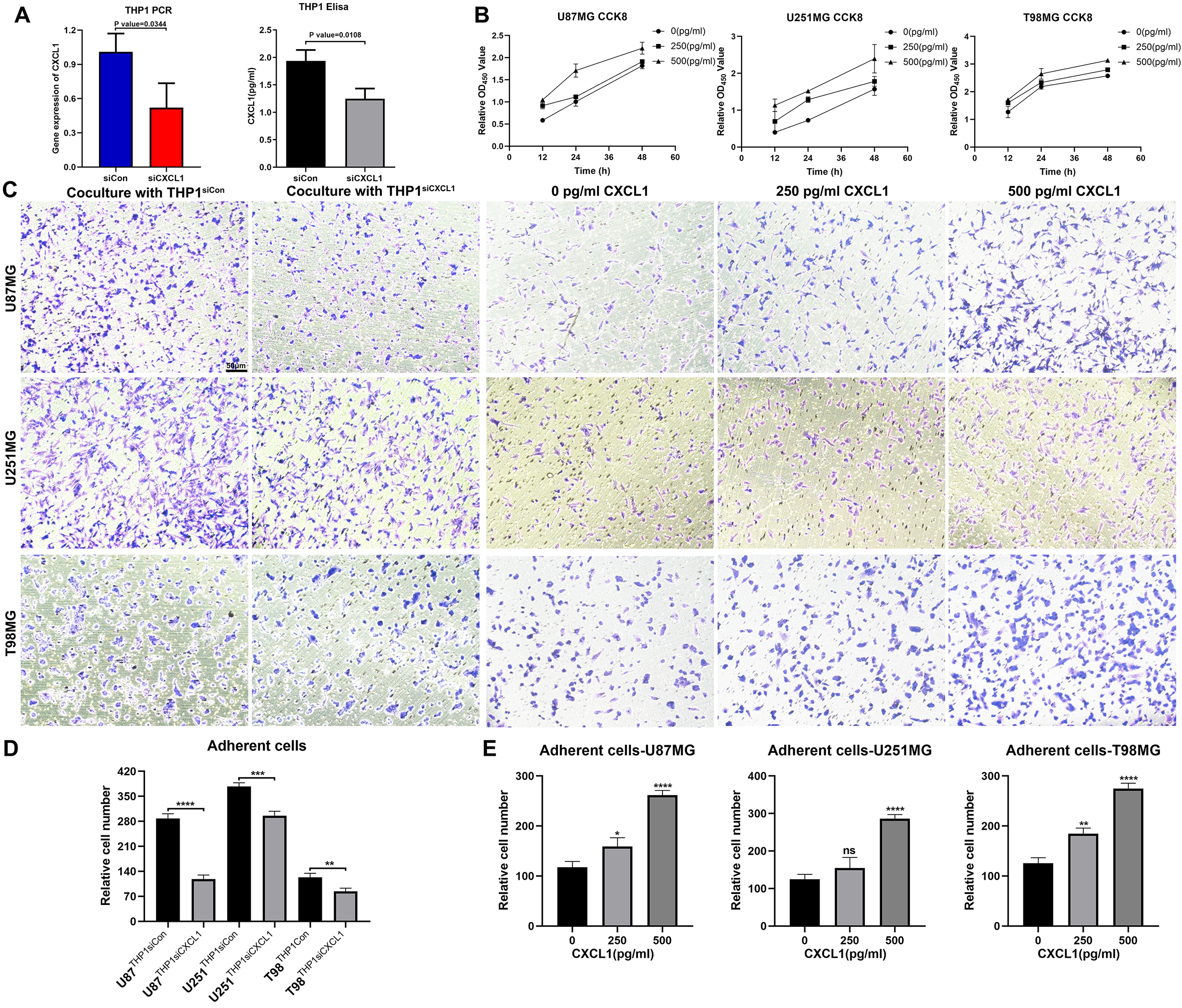

Supplement: Supplementary file 14 — sFigure 7 [file 41419_2025_7771_MOESM14_ESM.tif]

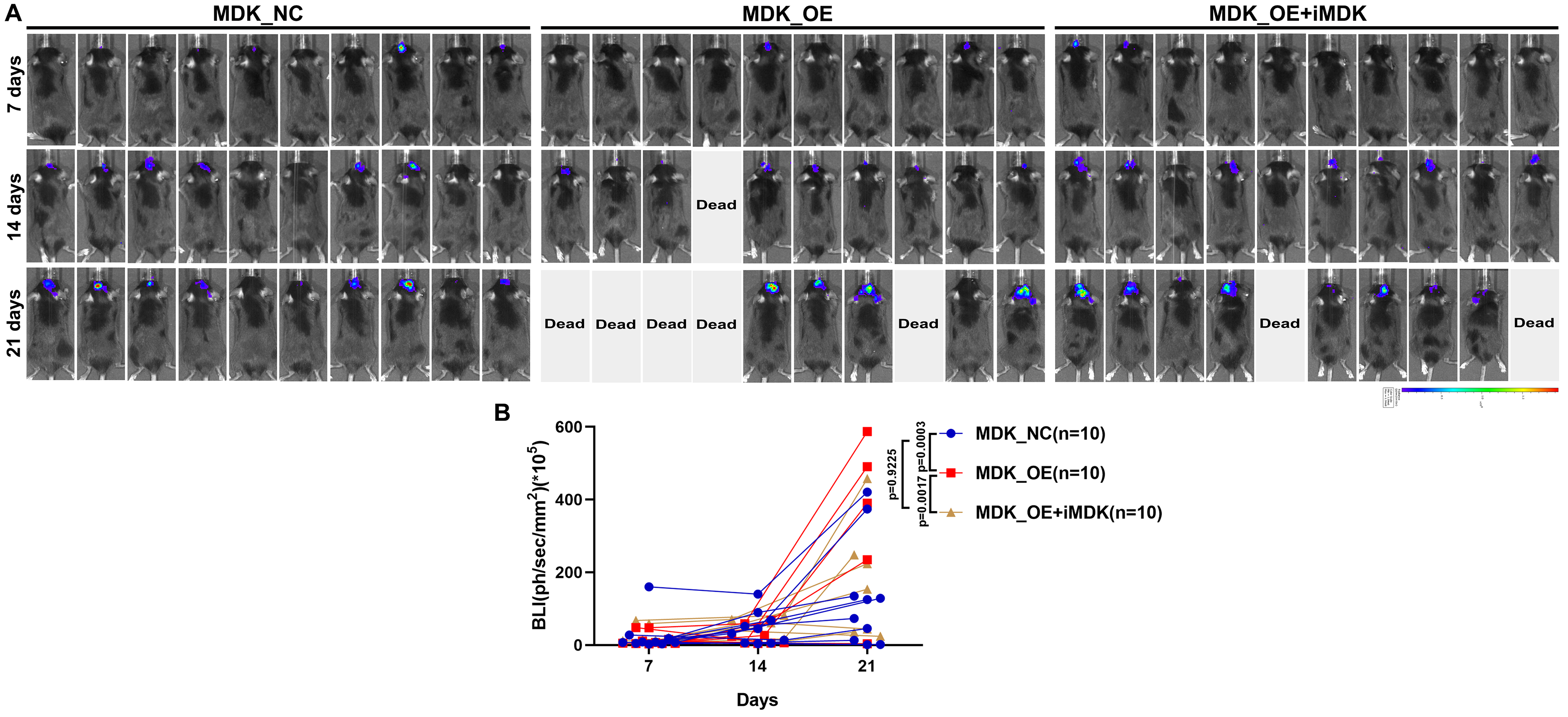

Supplement: Supplementary file 15 — sFigure 8 [file 41419_2025_7771_MOESM15_ESM.tif]

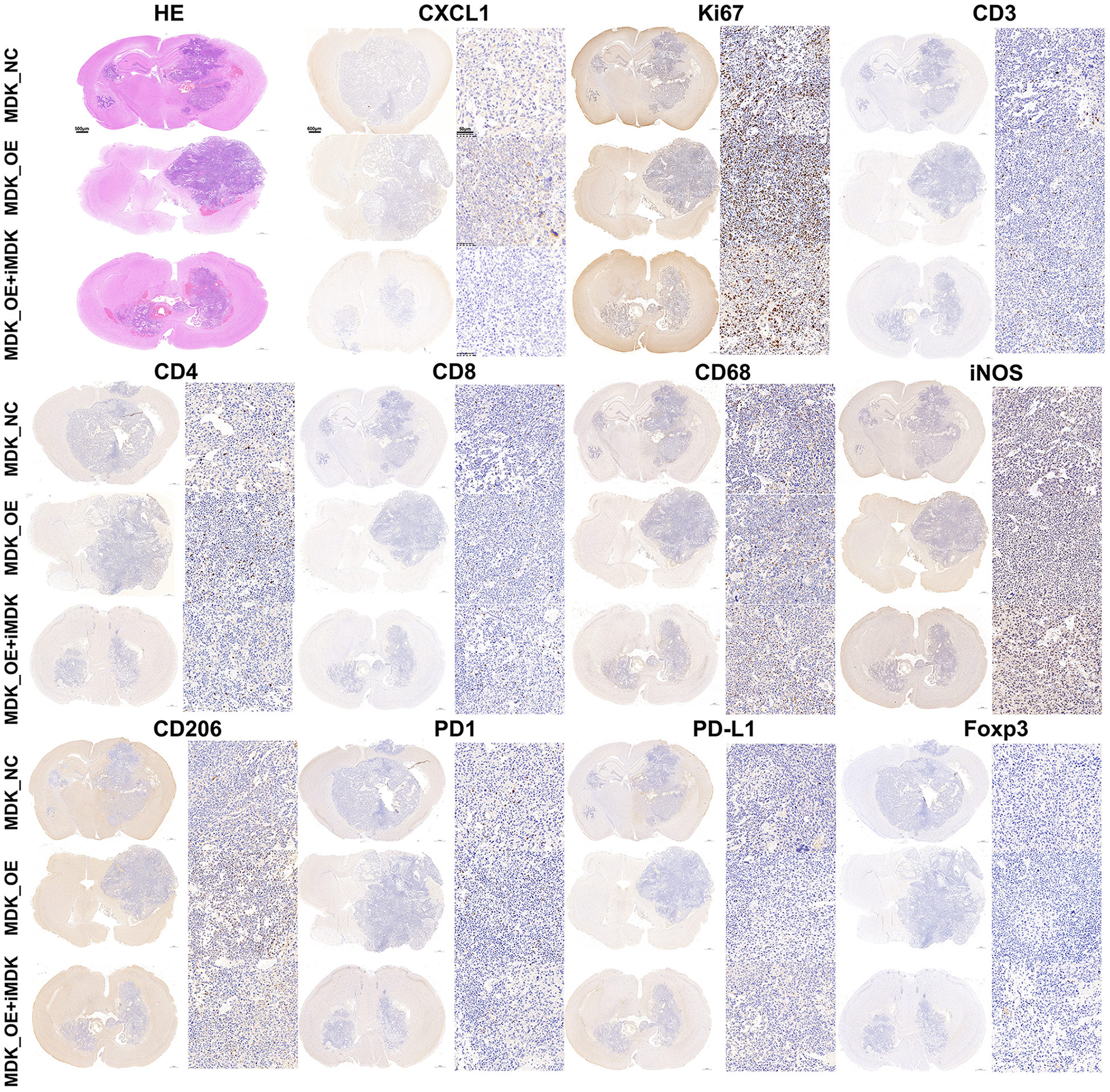

Supplement: Supplementary file 16 — sFigure 9 [file 41419_2025_7771_MOESM16_ESM.tif]

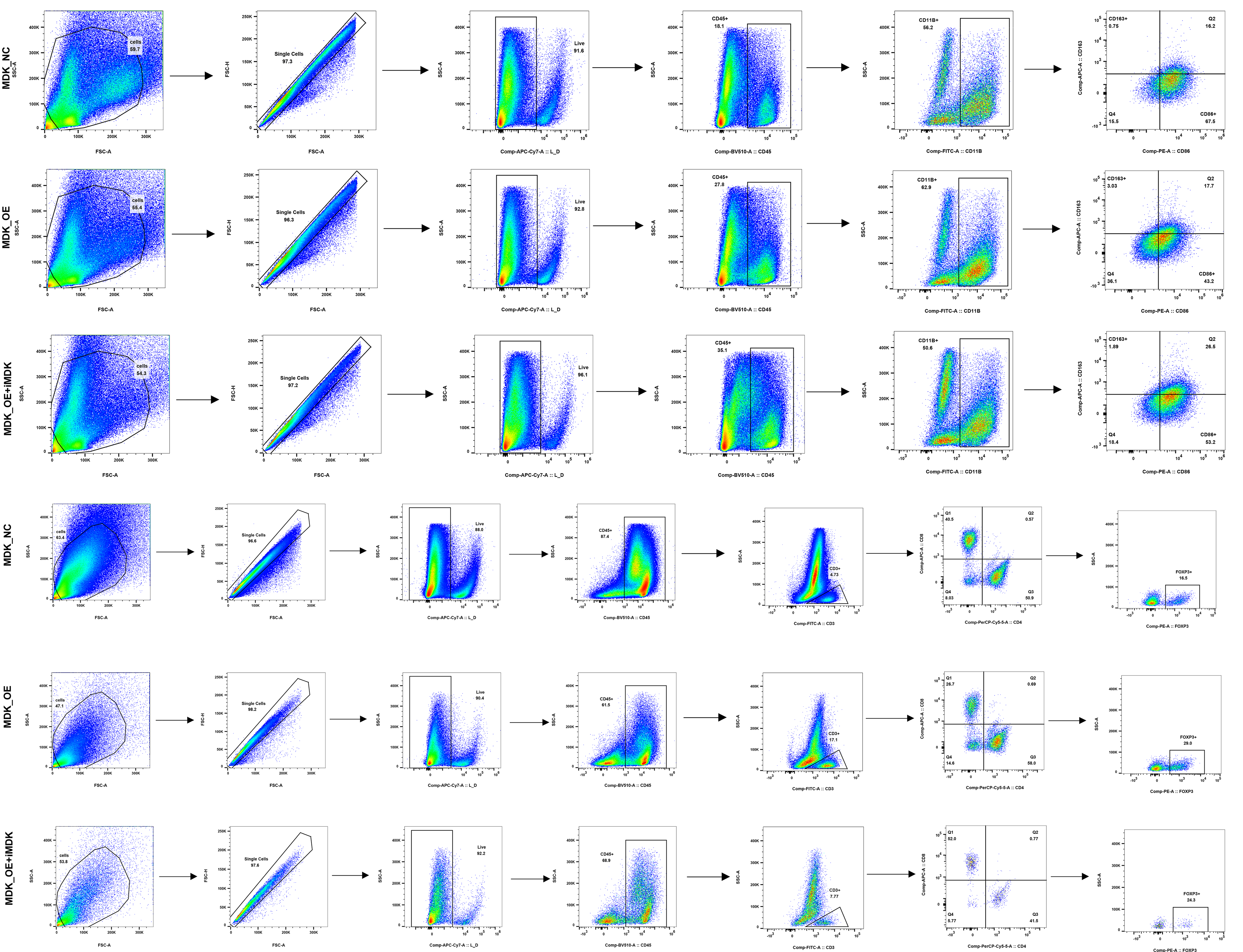

Supplement: Supplementary file 17 — sFigure 10 [file 41419_2025_7771_MOESM17_ESM.tif]
